# Species-specific bioluminescence facilitates speciation in the deep sea

**DOI:** 10.1007/s00227-014-2406-x

**Published:** 2014-02-21

**Authors:** Matthew P. Davis, Nancy I. Holcroft, Edward O. Wiley, John S. Sparks, W. Leo Smith

**Affiliations:** 1 University of Kansas, Lawrence, KS 66045 USA; 2 Johnson County Community College, Overland Park, KS 66210 USA; 3 American Museum of Natural History, New York, NY 10024 USA

## Abstract

The vast darkness of the deep sea is an environment with few obvious genetic isolating barriers, and little is known regarding the macroevolutionary processes that have shaped present-day biodiversity in this habitat. Bioluminescence, the production and emission of light from a living organism through a chemical reaction, is thought to occur in approximately 80 % of the eukaryotic life that inhabits the deep sea (water depth greater than 200 m). In this study, we show, for the first time, that deep-sea fishes that possess species-specific bioluminescent structures (e.g., lanternfishes, dragonfishes) are diversifying into new species at a more rapid rate than deep-sea fishes that utilize bioluminescence in ways that would not promote isolation of populations (e.g., camouflage, predation). This work adds to our understanding of how life thrives and evolution shaped present-day biodiversity in the deep sea, the largest and arguably least explored habitat on earth.

**Electronic supplementary material:**

The online version of this article (doi:10.1007/s00227-014-2406-x) contains supplementary material, which is available to authorized users.

## Introduction

Bioluminescence is the final product of a biochemical reaction whereby energy is converted to light following the breakdown of molecular bonds, typically the molecular decomposition of luciferin substrates by the enzyme luciferase in the presence of oxygen (Herring 1987; Haddock et al. 2010; Widder 2010). Bioluminescence has repeatedly evolved across the tree of life, from single-celled bacteria and dinoflagellates to fungi, jellyfishes, insects, and vertebrates (Herring 1987; Haddock et al. 2010; Widder 2010). Among animals, bioluminescence is used to communicate, defend against predation, and find or attract prey (Herring 1987; Haddock et al. 2010; Widder 2010). In some cases (e.g., fireflies, ostracods), unique bioluminescent signals have been hypothesized to aid in the process of speciation, with species recognition providing a mechanism to promote reproductive isolation among populations (Palumbi 1994; Branham and Greenfield 1996). In these bioluminescent organisms, the animals broadcast their identity with distinct light patterns. Among vertebrate lineages, bioluminescence has evolved only in cartilaginous and bony fishes that inhabit marine environments, with more than 80 % of luminous vertebrates confined to the deep sea (Herring 1987; Haddock et al. 2010).

Within teleosts, the production and emission of light is predominantly generated endogenously (e.g., the photophores of hatchetfishes and lanternfishes; Herring 1987; Haddock et al. 2010; Kronstrom and Mallefet 2010; Widder 2010), or through bacterially-mediated symbiosis (e.g., most anglerfish lures, flashlightfish subocular organs; Herring 1987; Dunlap et al. 2007; Haddock et al. 2010; Widder 2010). The functional utility of bioluminescence in a marine environment is both fascinating and wildly diverse, with incredible morphological specializations ranging from elongate species-specific barbels and lures to complex arrangements of photophores that are used to aid camouflage, defense, predation, and communication (Herring 1987; Haddock et al. 2010; Widder 2010). The utility of luminescence for predation in fishes ranges from red searchlights in the loosejaw dragonfishes *Malacosteus* (Douglas et al. 1998), to modified dorsal-fin spines in ceratioid anglerfishes that are used to lure in unsuspecting prey (Herring 2007). Camouflage and defensive strategies have repeatedly evolved across deep-sea marine lineages, including ventral counter-illumination, whereby an organism utilizes their bioluminescent photophores to match the intensity of downwelling light in an attempt to hide their silhouette from predators lurking below (Hastings 1971). Some bioluminescent organisms are even hypothesized to utilize light for intraspecific communication, including sexual selection (ponyfishes; Sparks et al. 2005; Chakrabarty et al. 2011) and species recognition (fireflies; Branham and Greenfield 1996).

The open ocean is an environment with few reproductive isolating barriers (Palumbi 1994) and comparatively low levels of species richness across different marine environments. Low species diversity in this environment is attributed to a general lack of physical barriers to gene flow and broad sympatry of populations (Palumbi 1994). Few studies have investigated patterns and mechanisms of diversification in the deep sea, and long-term behavioral studies are limited by the extreme conditions of the habitat (e.g., pressure, temperature). Lanternfishes (Myctophidae) are among the most species-rich families of marine teleosts and are endemic to this deep-sea, open-ocean environment (Eschmeyer 2013). Lanternfishes are consistently among the most abundant marine vertebrates collected around the globe in mid- to deep-water trawls (Sutton et al. 2010). The other predominant bioluminescent teleosts in this environment are the bristlemouths (Gonostomatidae: *Cyclothone*, *Gonostoma*, and *Sigmops*), which are estimated to be the most abundant vertebrates on earth (Sutton et al. 2010). Other common mesopelagic taxa include the marine hatchetfishes (Sternoptychidae) and dragonfishes (Stomiidae) (Sutton et al. 2010). Whereas bristlemouths (Gonostomatidae), overwhelmingly *Cyclothone*, have a strikingly higher biomass and account for more than 50 % of the total vertebrate abundance in mid-water habitats (~100–1,000 m), lanternfishes (Myctophidae) are the second most abundant family in this realm (Sutton et al. 2010). Interestingly, lanternfishes have diversified into 252 species, whereas only 21 species of bristlemouths have been described worldwide (Eschmeyer 2013). Bristlemouths and lanternfishes are superficially similar in size and body plan, with both groups possessing ventrally oriented bioluminescent photophores that provide camouflage, via ventral counter-illumination, against predation. However, lanternfishes have evolved an incredible array of modifications to their light-organ systems (photophores laterally on the body, sexually dimorphic luminescent organs on the tail or head) (Paxton 1972) that may have a significant impact on the evolutionary history and diversification of these fishes.

We investigate the mechanisms by which bioluminescence may directly contribute to reproductive isolation and diversification by studying the evolution of the photophore patterns, both lateral and ventral, in lanternfishes. To study this evolutionary phenomenon, we synthesize information from a temporal hypothesis of lanternfish evolutionary relationships, rates of teleostean fish diversification, photophore character evolution, and quantitative morphological data regarding the bioluminescent photophore system of lanternfishes to provide new insights into how this bioluminescent system might affect genetic isolation and diversification.

## Materials and methods

### Temporal phylogeny reconstruction, character evolution of bioluminescent structures, and estimates of species richness and diversification rate

**Fig. 1:**
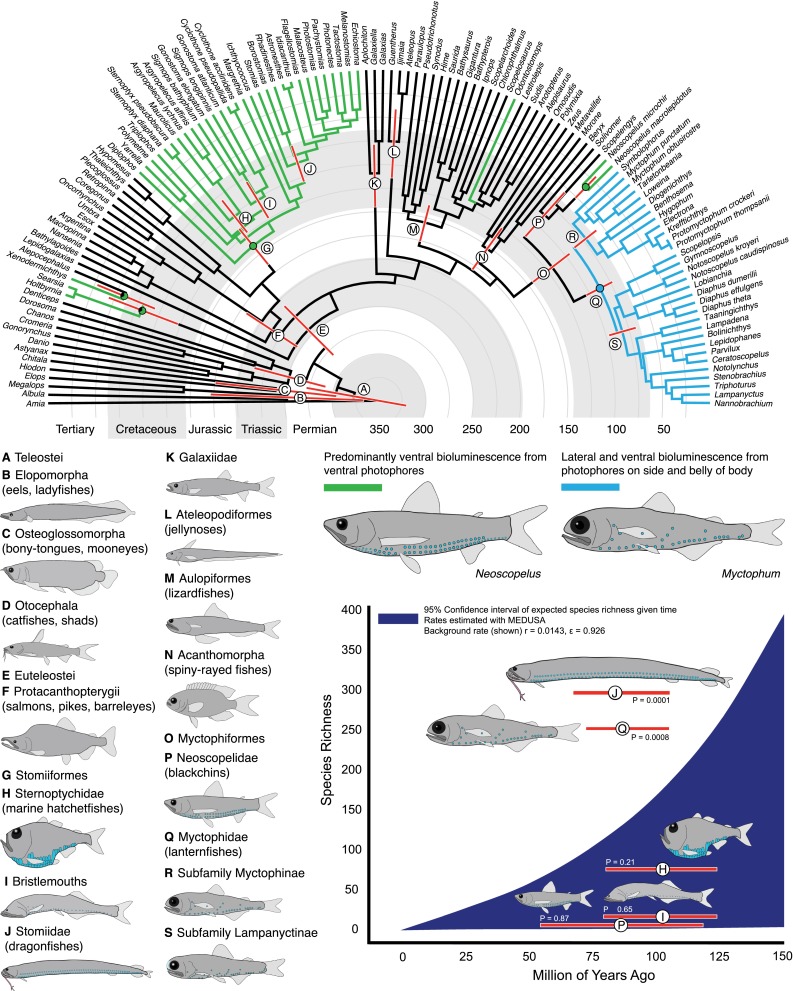
Temporal hypothesis of the relationships of ray-finned fishes indicating the evolution of bioluminescent photophores among representative deep-sea lineages. Species richness curve indicates the 95 % confidence interval for the expected number of species given clade age given a net diversification rate and relative rate of extinction

To assess the evolution of bioluminescent photophores that are found on the body of lanternfishes and the rate of lanternfish diversification through time, we inferred a temporal phylogeny of teleostean fishes from nuclear (RAG1, 1,479 bp; zic1, 895 bp) and mitochondrial (COI, 812 bp) data that incorporated six fossil calibrations with a lognormal prior (see below) and robust molecular taxonomic sampling of Myctophiformes from the families Neoscopelidae (blackchins, 3 of 3 genera) and Myctophidae (lanternfishes, 26 of 33 genera). The data matrix analyzed herein is 89.5 % complete, and sequences were aligned with MAFFT (Katoh et al. 2002) with taxon sampling listed in ESM table 1. Topology reconstruction and relative divergence times were estimated simultaneously under a relaxed uncorrelated lognormal clock using BEAST v.1.7.5 (Drummond and Rambaut 2007). Gene and codon positions were assigned models of nucleotide substitution from Akaike information criteria tests in jMODELTEST (Posada 2008), including GTR + G (COI position 2, zic1 position 2, zic1 position 3), and GTR + I + G for the remaining six codon positions. Four independent runs were performed with 100 million generations and a burnin of 15 million generations each. No topology constraints were enforced. Trees were sampled every 10,000 iterations, for a total of 40,000 trees, 34,000 post-burnin. Parameters and tree topologies converged on a stationary distribution and were visualized in TRACER v.1.5 (Rambaut and Drummond 2007). A 50 % maximum clade credibility (mean heights) tree was generated from the posterior tree distribution (Fig. 1).

Six fossil calibrations were assigned a lognormal prior, with hard minimum ages based on the oldest known fossil of the respective lineages. For further detailed accounts of fossil information, please refer to the previous studies listed below that also included the calibration.

Holostei + Teleostei (Near et al. 2012) (C1): The age of †*Watsonulus eugnathoides*, a stem-lineage Halecomorphi was used to denote a minimum age of 245.9 Ma for the common ancestor of Holostei + Teleostei, with a conservative soft upper bound set to 325 Ma based on the minimum age of †*Cosmoptychius striatus*, a crown group of Actinopteri. Prior settings included a mean of 2.411 and a standard deviation of 1.0.

Chanidae (Near et al. 2012) (C2): The age of †*Rubiesichthys gregalis*, a stem lineage of the family Chanidae, was used to date a minimum age for the most recent common ancestor of *Chanos* and *Cromeria* to 133.9 Ma. A soft upper bound of 150.8 Ma was designated based on the stem-lineage ostariophysian †*Tischlingerichthys viohi*. Prior settings included a mean of 0.87 and a standard deviation of 1.0.

Euteleostei (Davis and Fielitz 2010) (C3): The age of †*Leptolepides sprattiformis*, a stem-lineage euteleost, was used to date the most recent common ancestor of Euteleostei and Otocephala at a minimum of 150 Ma. A soft upper bound of 220 Ma was set based on the age of the stem-lineage teleost †*Pholidophorus bechei*. Prior settings included a mean of 2.289 and a standard deviation of 1.0.

Alepisauridae (Davis and Fielitz 2010) (C4): The age of †*Enchodus brevis*, a extinct species that is identified in a clade that is the sister group to extant alepisaurid taxa (*Alepisaurus* and *Omosudis*), was used to date the minimum age of the most recent common ancestor of Alepisauridae to 100 Ma. A conservative soft upper bound was set to 150 Ma, the age of the oldest known stem-lineage euteleost †*Leptolepides sprattiformis*. Prior settings included a mean of 1.953 and a standard deviation of 1.0.

Myctophiformes (Davis and Fielitz 2010) (C5): The stem-lineage myctophiform †*Sardiniodes*, known from Late Cretaceous Middle Cenomanian deposits in the Hakel and Hanjula formations, was used to date the minimum age of the most recent common ancestor of Myctophiformes + Acanthomorpha at 96 Ma. A conservative soft upper bound was set to 150 Ma, the age of the oldest known fossil euteleost †*Leptolepides sprattiformis*. Prior settings included a mean of 2.029 and a standard deviation of 1.0.

Acanthomorpha (Near et al. 2012) (C6): The minimum age of the most recent common ancestor of Acanthomorpha was conservatively dated to a minimum age of 93.6 Ma, based on the fossil polymixiiform taxa †*Homonotichthys dorsalis*. A conservative soft upper bound was set to 150 Ma, the age of the oldest known fossil euteleost †*Leptolepides sprattiformis*. Prior settings included a mean of 2.7072 and a standard deviation of 1.0.

Likelihood-based ancestral character state reconstruction was performed in Mesquite 2.7 (Maddison and Maddison 2002; Fig. 1, ESM Fig. 1). The GEIGER module within the R platform was used to calculate a 95 % confidence interval of the expected number of species within a clade given a net diversification rate (*r*), a relative extinction rate (*ε*), and clade age (Magallón and Sanderson 2001; Harmon et al. 2008). Rates for net diversification and relative extinction were estimated with MEDUSA (ESM Fig. 1) in R using the GEIGER module (Harmon et al. 2008; Alfaro et al. 2009), with species richness numbers including the amount of currently valid described species for each group identified with the *Catalog of Fishes* resource (Eschmeyer 2013).

### Geometric morphometric relative warp analyses of bioluminescent photophore positions

Digital images of photophore patterns were taken with a Canon EOS Digital Rebel 10.1 megapixel camera, with material examined listed in ESM table 2. Digital Landmarks were assigned with software tpsDIG v.2.16 (Rohlf 2010a) following a standardized procedure of placing landmarks directly on the positionally and structurally homologous photophores being examined (Ray 1950; Paxton 1972). Landmark configurations were rotated, scaled, and aligned to a unit centroid size through generalized procrustes analysis (Rohlf and Slice 1990) with the consensus configuration as a starting point. Relative warp analysis was done using the software tpsRelw v1.49 (Rohlf 2010b), which quantifies the change in shape of each individual from the consensus configuration, and is a principal component analysis.

## Results

### Phylogeny reconstruction and character evolution of bioluminescent structures

Our phylogeny (Fig. 1, ESM Fig. 1) recovers Myctophiformes as the sister group of the spiny-rayed fishes (Acanthomorpha), which is consistent with previous morphological (Rosen 1973) and molecular (Davis and Fielitz 2010; Near et al. 2012) work. The two families of Myctophiformes are sister groups and monophyletic (Fig. 1), including the species-rich lanternfishes (Myctophidae, 252 species in 33 genera) and the comparatively depauperate blackchins (Neoscopelidae, 6 species in three genera). Maximum likelihood ancestral character state reconstructions indicate that the common ancestor of Myctophiformes lacked bioluminescent photophores (Fig. 1, clade O), which is consistent with the known fossil record of stem-lineage Myctophiformes (Goody 1969), and our temporal hypothesis indicates that the myctophiforms most likely first diversified sometime during the Early-Late Cretaceous, between 144 and 95 million years ago (Fig. 1, clade O). Within the blackchins (Neoscopelidae, Fig. 1, clade P), bioluminescent structures evolved in the common ancestor of the genus *Neoscopelus*, with photophores restricted to the ventral surface of the body and developing underneath each individual ventral scale (Paxton 1972). Bioluminescent structures are absent in the other blackchin genera (*Scopelengys* and *Solivomer*). The functional role of bioluminescence in blackchins is likely ventral counter-illumination, which is hypothesized to be the principle function of camouflage for the majority of bioluminescent mesopelagic vertebrates in the water column (Hastings 1971).

### Species richness and diversification

Models for diversification rates across Actinopterygii were estimated using MEDUSA (Alfaro et al. 2009), which identified a model incorporating three significant rate shifts as a better fit across ray-finned fishes (ΔAIC score greater than 4 as recommended by Alfaro et al. 2009) than a model of evolution that incorporated no shifts in diversification (ESM Fig. 1). The MEDUSA analysis estimated a net background diversification rate (*r*) for Actinopterygii of 0.0143 with a relative rate of extinction (*ε*) of 0.926 (Fig. 1, ESM Fig. 1). Rate shifts included two rate increases in the Ostariophysi (*r* = 0.0386, *ε* = 0.62) and Acanthomorpha (*r* = 0.0516, *ε* = 0.647) and a rate decrease in the monotypic family Lepidogalaxiidae (*r* = 008e−14, *ε* = 0.01).

The background diversification rates estimated from MEDUSA were used to calculate a 95 % confidence interval of the expected number of species within a clade for the bioluminescent lineages investigated herein. Lanternfishes (Myctophidae, 252 species) that have lateral species-specific photophore patterns exhibit exceptional species diversity given clade age (Fig. 1, clade Q), whereas the blackchins (Neoscopelidae, 6 species), which lack lateral body photophores, fall well within the 95 % confidence interval of expected species diversity given clade age (Fig. 1, clade P). Other clades of meso-bathypelagic deep-sea fishes that utilize ventral bioluminescence primarily for camouflage, such as the bristlemouths (Fig. 1, clade I, 25 species) and marine hatchetfishes (Fig. 1, clade H, 75 species), were also identified as not being exceptionally species-rich given clade age. In contrast, the stomiid dragonfishes, which have species-specific bioluminescent barbels (Sutton and Hartel 2004), were found to exhibit exceptional species diversity given clade age (Fig. 1, clade J, 294 species).

### Relative warp analyses of bioluminescent photophore position

**Fig. 2:**
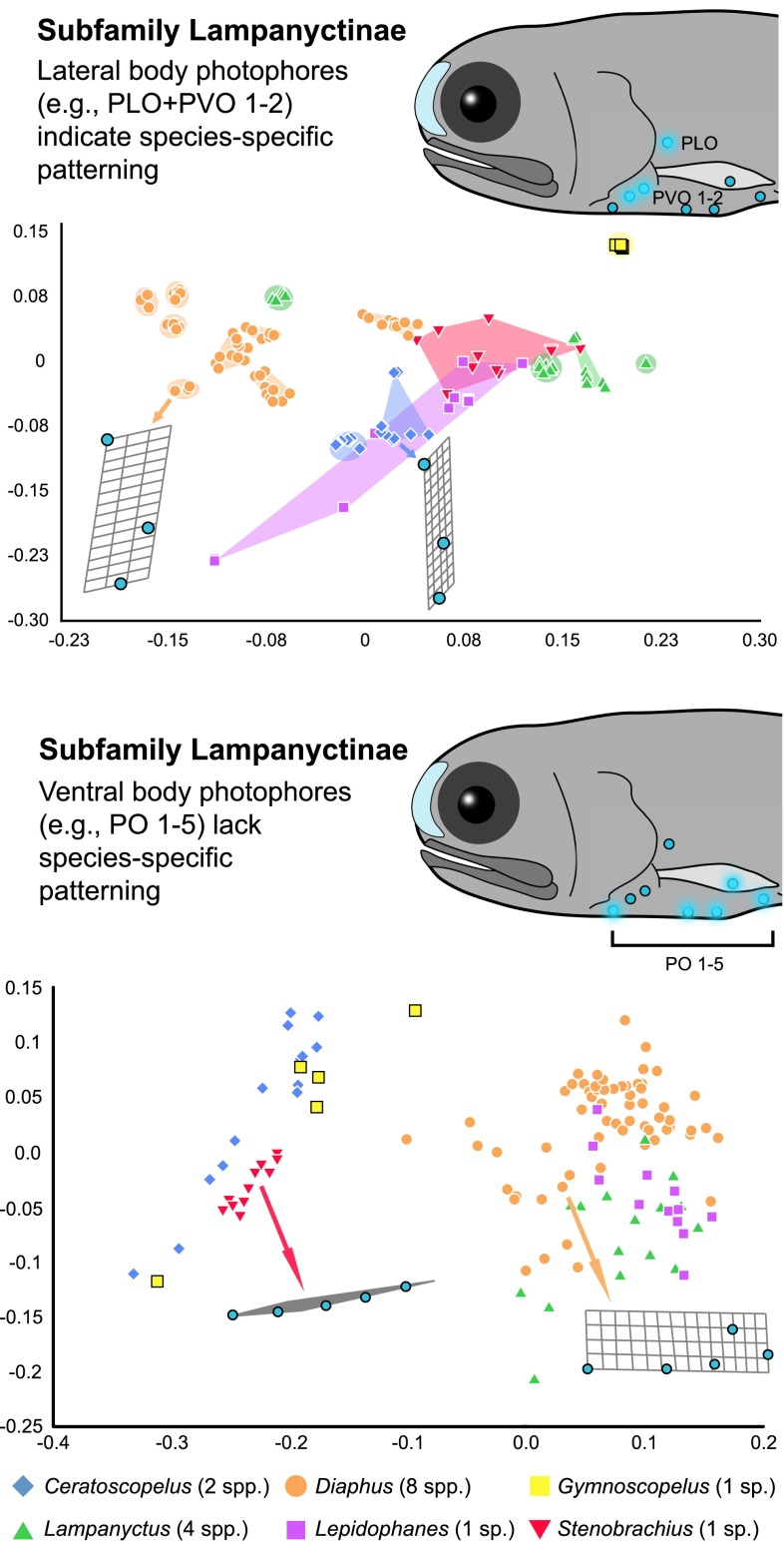
Relative warp analysis of lateral and ventral photophore series among genera in the lanternfish subfamily Lampanyctinae for the first (*X* axis) and second (*Y* axis) relative warp scores

**Fig. 3:**
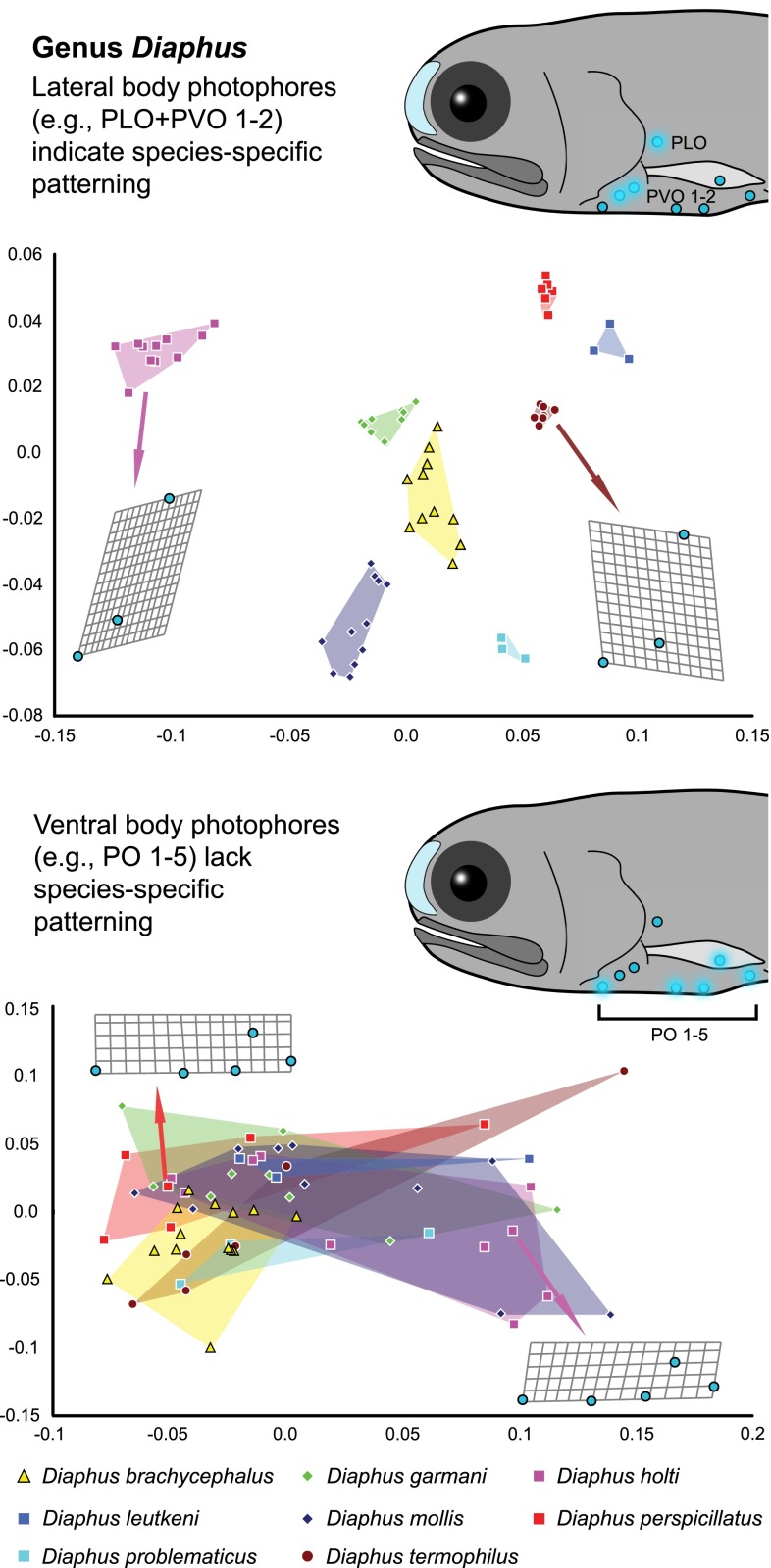
Relative warp analysis of lateral and ventral photophore series among species in the lanternfish genus *Diaphus f*or the first (*X* axis) and second (*Y* axis) relative warp scores

The distribution of photophores on the lateral sides of the body (ESM Fig. 2; i.e., PLO + PVO series, SOA series, and PRC series) can be used to differentiate species across genera (Fig. 2, ESM Fig. 3) and within a genus (Fig. 3, ESM Fig. 3). Across genera within the lanternfish subfamilies Myctophinae (ESM Fig. 3) and Lampanyctinae (Fig. 2), we note that often when one species has a similar photophore pattern quantitatively to another species for a specific series (e.g., PLO + PVO), they were often from different genera (Fig. 2, ESM Fig. 3) and/or these lineages included additional species delimitating variation in the other lateral photophore patterns investigated (ESM Fig. 3).

Overall, species-specific delimitation is more obscured for the ventrally-oriented photophores (VO, PO series, ESM Fig. 2) among species within a genus, such as *Diaphus* (Fig. 3, ESM Fig. 3). When taxa from different lampanyctine genera were compared, the distribution of ventral photophores among species from the same genus is more similar to each other than to species in other genera (Fig. 2, ESM Fig. 3), with these differences often corresponding to varying degrees of dorsal displacement of one of the ventral photophores. Taxa within the subfamily Myctophinae exhibited fewer instances of dorsal displacement in these ventral series, leading to more significant overlap across genera (ESM Fig. 3). The lampanyctine genus *Lampanyctus* is one case observed here where a ventral series indicated isolated species clusters; however, this clustering is associated with differences in the amount of dorsal displacement of a single photophore (VO 2) that has migrated further up the body in some species (ESM Fig. 3S, VO series).

## Discussion

### Evolution of bioluminescent photophore system in lanternfishes

Unlike most marine fishes that have bioluminescent photophores, lanternfishes (Myctophidae) also have numerous lateral photophores on the sides of their bodies. The lateral photophores have been consistently hypothesized to be homologous structures based on spinal nerve innervations, with these photophores having “migrated” dorsally through time from photophores in a common ancestor that lined the ventral surface of the fish (Tåning 1918; Parr 1928; Bolin 1939; Fraser-Brunner 1949; Ray 1950). Our phylogeny and ancestral character reconstruction indicate that migration of some ventral photophores dorsally onto the body most likely began in the common ancestor of the lanternfishes (Myctophidae), approximately 104–73 million years ago during the Late Cretaceous (Fig. 1, clade Q). Whereas the ventral photophores that line the underside of myctophid lanternfishes have long been considered to provide camouflage against predation in their open-ocean environment, the functional significance of the lateral body photophores has remained obscure. Previous studies have suggested that the placement of these homologous lateral body photophores creates taxonomically useful patterning and may be used to infer the evolutionary relationships among lanternfish lineages (Tåning 1918; Parr 1928; Bolin 1939; Fraser-Brunner 1949; Paxton 1972); however, the observed differences among species within a particular genus were considered potentially slight enough as to not play a critical role in species recognition, although the remarkable species richness of the group coupled with the diversity of photophore patterning is in need of further macroevolutionary study (Paxton 1972).

To investigate the potential for lateral body photophores to contribute to species recognition, we examined the photophore patterns of myctophid lanternfishes that are conserved across taxa in both number and position, and are hypothesized to be homologous structures based on positional and structural homology criteria (Ray 1950; Remane 1971; Paxton 1972). These photophore patterns were examined quantitatively using landmark-based geometric morphometric techniques (Figs. 2, 3, ESM Figs. 2, 3). We found that there is sufficient variation in photophore placement on the lateral sides of the body among genera (Fig. 2, ESM Fig. 3) and among species within a genus, to quantitatively separate and identify species (Fig. 3, ESM Fig. 3). These findings indicate that the lateral body photophores within the species-rich lanternfishes (Myctophidae, 252 species) exhibit species-specific properties that may play a functional role in species recognition, and, as a result, contribute to genetic isolation within their deep-sea (predominantly found from 100 to 1,000 m; Sutton et al. 2010), open-ocean habitat.

In contrast to the lateral photophores of the lanternfish light-organ system, we identified little to no species-specific properties in the ventral photophore patterns examined herein (PO series, VO series, Figs. 2, 3, ESM Fig. 3). Other ventral photophores, such as the AOa and AOp series that cover the ventral surface on the caudal end of the fish (ESM Fig. 2), often vary in number among individuals within a single species. Overall, our result of significantly less species-specific patterning within a genus for the ventral photophores is consistent with what we would expect if the primary function of these ventral structures is camouflage and not species recognition. Our results corroborate the previous hypothesis that the primary function of the predominantly ventral photophore system is most likely for camouflage and predator avoidance (e.g., Tåning 1918; Parr 1928; Bolin 1939; Fraser-Brunner 1949; Ray 1950) with the evolution of species-specific lateral body photophore patterns occurring secondarily. A similar pattern has been observed in terrestrial organisms, such as fairy-wren (*Malurus*) birds, where song-like trills are hypothesized to have evolved initially for predator avoidance, with species-specific trills secondarily evolving across the lineage (Greig and Webster 2014).

Studies regarding visual acuity (Douglas and Partridge 1997; Douglas et al. 2002, 2003; Turner et al. 2009) and eye-size variability (Busserolles et al. 2013) in lanternfishes indicate that they are physiologically capable of visualizing bioluminescent patterns and emissions in their deep-sea environment. Studies on the visual pigment absorption maxima of lanternfishes have shown that the possession of a retina with a single pigment capable of absorbing bioluminescent light is taxonomically widespread across lanternfishes, with light absorption ranging from 480 to 492 nm in species from both subfamilies Myctophinae and Lampanyctinae (Turner et al. 2009). Lanternfishes are hypothesized to detect this blue-green bioluminescent light at a distance of up to 30 m in their deep-sea environment (Turner et al. 2009). The work presented herein compliments these earlier studies on the visualization system of lanternfishes and suggests that in addition to having the visual acuity to observe bioluminescent light in their environment at considerable distance, lanternfishes also possess species-specific bioluminescent emission patterns that could play an important role in species recognition and genetic isolation within a deep-sea habitat.

### Patterns of diversification within Myctophiformes (blackchins and lanternfishes)

**Fig. 4:**
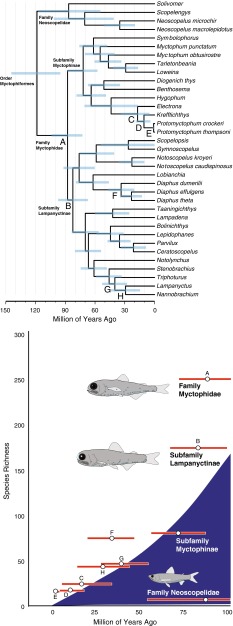
Temporal hypothesis of the relationships of the Myctophiformes (Neoscopelidae and Myctophidae). Species richness curve indicates the 95 % confidence interval for the expected number of species given clade age given a net diversification rate and relative rate of extinction

If the evolution of species-specific bioluminescence patterns are a driving force behind genetic isolation in the deep sea for lanternfishes, as they are hypothesized to be for other groups using bioluminescence for communication (e.g., fireflies; Branham and Greenfield 1996), they may exhibit a greater than expected species richness given their clade age. Our phylogenetic analysis indicates that while the sister clades of blackchins (Neoscopelidae, 6 species) and lanternfishes (Myctophidae, 252 species) initially diverged at similar times within the Late Cretaceous (Figs. 1, 4), the tempo and mode of their diversification differ considerably (Figs. 1, 4). Bioluminescent structures among the depauperate blackchin taxa are either wholly absent (*Scopelengys*, *Solivomer*) or restricted to ventrally-oriented photophores (*Neoscopelus*) where the primary function is hypothesized to be for counter-illumination (Hastings 1971). In contrast, we identified that lanternfishes (Myctophidae), with species-specific bioluminescent structures, exhibit exceptional species diversity given their estimated clade age (Figs. 1, 4). As discussed earlier and quantitatively identified in this study, myctophid lanternfishes have evolved a complex species-specific photophore system, in addition to other bioluminescent structures (e.g., caudal light organs, suborbital light organs), which have been observed to be morphologically variable, species specific, occasionally sexually dimorphic, and taxonomically widespread throughout the lanternfish radiation (Paxton 1972).

Among the species-rich lanternfishes (Myctophidae), lineages within the subfamily Lampanyctinae are inferred to be diversifying at a higher rate than those within the subfamily Myctophinae (Fig. 4). In regard to bioluminescent structures, species within Lampanyctinae differ from those in Myctophinae in possessing some bioluminescent lateral body photophores that have extensively migrated dorsally to near (e.g., *Lampanyctus*) or even above (e.g., *Triphoturus*) the lateral line (Paxton 1972). Myctophine taxa additionally lack any secondary photophores or photophores on the cheek (Paxton 1972) and are restricted to two or fewer Prc (ESM Fig. 2) caudal photophores, whereas Lampanyctinae taxa may possess anywhere from 3 to 9 Prc caudal photophores. Within Myctophinae, a clade comprising the genera *Electrona*, *Krefftichthys*, and *Protomyctophum* was identified as having exceptional species richness given clade age.

Within Lampanyctinae, our results indicate that the genus *Diaphus* (76 species) exhibits exceptional species diversity given clade age (Fig. 4). Many species within *Diaphus* are notable for having enlarged suborbital light organs and luminous patches on the body (Bolin 1959), with the anterior light organs being sexually dimorphic in some species (Paxton 1972). In addition to the species-specific lateral body photophores, the orbital organs of *Diaphus* have distinctive species-specific anatomy (Nafpaktitis 1968). The evolution of bioluminescent orbital organs, which are species-specific and sexually dimorphic, has likely functioned as a further mechanism toward genetic isolation that has impacted the diversification of the *Diaphus* lineage. These anterior luminescent structures likely serve additional functions as well, including predation and defensive predator avoidance. Other lampanyctine clades that indicated exceptional species richness given clade age include the *Lampanyctus* + *Nannobrachium* (42 species) clade, a radiation of lanternfishes in which species possess additional photophores on their cheek, and secondary photophores on the head and body near the lateral line (Paxton 1972).

### Evolution of bioluminescent structures and diversification in the deep sea

As a result of diversifying in an environment with few reproductive isolating barriers (Palumbi 1994), lineages of deep-sea fishes that functionally use bioluminescence for species recognition as a mechanism of reproductive isolation are likely to be more diverse than other deep-sea lineages where bioluminescence is primarily used for other functions (e.g., camouflage, prey detection or attraction). Among the deep-sea lineages examined here with bioluminescent structures, we identified that lineages with species-specific anatomical bioluminescent structures that may serve species recognition functions were found to exhibit exceptional species diversity given clade age (Figs. 1, 4). While they occupy the same general marine habitat and are estimated to have diverged during similar times in the Late Cretaceous, lanternfishes (Myctophidae, 252 species) with species-specific bioluminescent photophore patterns and structures (e.g., caudal light organs) are exceptionally species-rich given clade age when compared to open-ocean lineages with ventrally oriented photophores, including the bristlemouths (Gonostomatidae, 21 species: *Cyclothone*, *Gonostoma*, and *Sigmops*), which are incredibly abundant with regards to biomass in the open ocean (Sutton et al. 2010), marine hatchetfishes (Sternoptychidae, 75 species), and blackchins (Neoscopelidae, 6 species) as indicated in Fig. 1. Within the Stomiiformes, dragonfishes (Stomiidae, 294 species) were also found to exhibit exceptional species diversity given clade age (Figs. 1, 4). The majority of dragonfish species possess bioluminescent chin barbels that are anatomically distinct across species and are frequently a key diagnostic feature used to identify and describe species (e.g., Sutton and Hartel 2004). These results are further evidence that species-specific bioluminescent structures and/or photophore patterns may provide a mechanism of genetic isolation in deep-sea fishes where bioluminescence may be playing a functional role in species recognition.

While our results indicate that deep-sea lineages with species-specific bioluminescent structures, which may have an important functional role in species recognition and genetic isolation, indicate higher than expected species richness given clade age, it is possible that additional factors have contributed to diversification in these lineages. For example, diversification in the lampanyctine lanternfishes may have also been driven by the elongation of the lower jaw observed in that clade. This morphological change may have led to new niche availability with bioluminescent reproductive isolation playing a more supportive role in their lineage accumulation over time. In general, it is difficult to disentangle all of the factors that may have led to the present-day species richness we observe in deep-sea fauna; however, if bioluminescence is playing a role in communication with regards to species recognition and sexual selection, it represents an important reproductive isolation mechanism in this fascinating marine environment.

### Conclusions

The results of this study indicate that the common ancestor of lanternfishes most likely evolved their complex body photophore system during the Late Cretaceous, and since this time lanternfishes have diversified into one of the most species-rich clades of meso-bathypelagic vertebrate lineages in oceans worldwide. We show, for the first time, using quantitative data, that the lanternfish photophore system most likely has two functional roles, one for camouflage from predators (ventral body photophores) and one for species recognition (lateral body photophores). We also show that the present-day diversity of lanternfishes is significantly greater than expected given clade age, whereas other bioluminescent meso-bathypelagic fish lineages in the same habitat that utilize bioluminescence predominantly for ventral counter-illumination (i.e., bristlemouths and marine hatchetfishes) do not exhibit greater than expected species richness (Fig. 1). This, coupled with our in-depth analysis of lanternfish photophore evolution and function, indicates that species-specific bioluminescent structures impact species recognition for deep-sea bioluminescent lineages, acting as a mechanism for genetic isolation in an open-ocean habitat that has few obvious genetic isolating barriers. The evolution of species-specific bioluminescent communication in lanternfishes indicates that bioluminescence may have direct impacts on speciation in an aquatic environment, much like it does for fireflies in a terrestrial habitat.

## Electronic supplementary material

Below is the link to the electronic supplementary material.

Supplementary material 1 (PDF 8849 kb)

## References

[CR1] Alfaro ME, Santini F, Brock C, Alamillo H, Dornburg A, Rabosky DL, Carnevale G, Harmon LJ (2009). Nine exceptional radiations plus high turnover explain species diversity in jawed vertebrates. Proc Natl Acad Sci.

[CR2] Bolin RL (1939). A review of the myctophid fishes of the Pacific coast of the United States and of Lower California. Stanf Ichthyol Bull.

[CR100] Bolin RL (1959) Iniomi. Myctophidae from the "Michael Sars" North Atlantic deep-sea expedition 1910. Rep Sci Res "Michael Sars" Deep-Sea Exped 4(7):1–45

[CR3] Branham MA, Greenfield MD (1996). Flashing males win mate success. Nature.

[CR4] Busserolles F, Fitzpatrick JL, Paxton JR, Marshall NJ, Collin SP (2013). Eye-size variability in deep-sea lanternfishes (Myctophidae): an ecological and phylogenetic study. PLoS ONE.

[CR5] Chakrabarty P, Davis MP, Smith WL, Baldwin Z, Sparks JS (2011). Is sexual selection driving diversification of the bioluminescent ponyfishes (Teleostei: Leiognathidae)?. Mol Ecol.

[CR6] Davis MP, Fielitz C (2010). Estimating divergence times of lizardfishes and their allies (Euteleostei: Aulopiformes) and the timing of deep-sea adaptations. Mol Phylogenet Evol.

[CR7] Douglas RH, Partridge JC (1997). On the visual pigments of deep-sea fish. J Fish Biol.

[CR8] Douglas RH, Partridge JC, Dulai K, Hunt D, Mullineaux CW, Tauber AY, Hynninen PH (1998). Dragon fish see using chlorophyll. Nature.

[CR9] Douglas RH, Bowmaker JK, Mullineaux CW, Stanley PE, Kricka LJ (2002). A possible retinal longwave detecting system in a myctophid fish without far-red bioluminescence; evidence for a sensory arms race in the deep-sea. Bioluminescence and chemiluminescence, progress and current applications.

[CR10] Douglas RH, Hunt DM, Bowmaker JK, Colin SP, Marshall NP (2003). Spectral sensitivity tuning in the deep-sea. Sensory processing in aquatic environments.

[CR11] Drummond AJ, Rambaut A (2007). BEAST: Bayesian evolutionary analysis by sampling trees. BMC Evol Biol.

[CR12] Dunlap PV, Ast JC, Kimura S, Fukui A, Yoshino T, Endo H (2007). Phylogenetic analysis of host-symbiont specificity and codivergence in bioluminescent symbioses. Cladistics.

[CR13] Eschmeyer WN (2013) Catalog of fishes. California Academy of Sciences. http://research.calacademy.org/research/ichthyology/catalog/fishcatmain.asp). Electronic version

[CR14] Fraser-Brunner A (1949). A classification of the fishes of the family Myctophidae. Proc Zool Soc Lond.

[CR15] Goody PC (1969). The relationships of certain upper cretaceous teleosts with special reference to the myctophoids. Bull Br Mus Nat Hist Geo.

[CR16] Greig EI, Webster MS (2014). How do novel signals originate? The evolution of fairy-wren songs from predator to display contexts. Anim Behav.

[CR17] Haddock SHD, Moline MA, Case JF (2010). Bioluminescence in the sea. Annu Rev Mar Sci.

[CR18] Harmon LJ, Weir JT, Brock CD, Glor RE, Challenger W (2008). GEIGER: investigating evolutionary radiations. Bioinformatics.

[CR19] Hastings JW (1971). Light to hide by: ventral luminescence to camouflage the silhouette. Science.

[CR20] Herring PJ (1987). Systematic distribution of bioluminescence in living organisms. J Biolumin Chemilumin.

[CR21] Herring PJ (2007). Sex with the lights on? A review of bioluminescent sexual dimorphism in the sea. J Ma Biol Assoc UK.

[CR22] Katoh K, Misawa K, Kuma K, Miyata T (2002). MAFFT: a novel method for rapid multiple sequence alignment based on fast Fourier transform. Nucl Acids Res.

[CR23] Kronstrom J, Mallefet J (2010). Evidence for widespread involvement of NO in control of photogenesis in bioluminescent fish. Acta Zool.

[CR24] Maddison WP, Maddison DR (2002) Mesquite: a modular system for evolutionary analysis. Version 2.7 http://mesquiteproject.org

[CR25] Magallón S, Sanderson MJ (2001). Absolute diversification rates in angiosperm clades. Evolution.

[CR26] Nafpaktitis BG (1968). Lanternfishes of the genera *Lobianchia* and *Diaphus* in the North Atlantic. Dana Rept.

[CR27] Near TJ, Eytan RI, Dornburg A, Kuhn KL, Moore JA, Davis MP, Wainwright PC, Friedman M, Smith WL (2012). Resolution of ray-finned fish phylogeny and timing of diversification. Proc Natl Acad Sci.

[CR28] Palumbi SR (1994). Genetic divergence, reproductive isolation, and marine speciation. Annu Rev of Ecol Syst.

[CR29] Parr AE (1928). Deepsea fishes of the order Iniomi from the water around the Bahama and Bermuda Islands, with annotated keys to the Sudidae, Myctophidae, Scopelarchidae, Evermannellidae, Omosudidae, Cetomimidae and Rondeletiidae of the world. Bull Bingham Oceanogr Coll.

[CR30] Paxton JR (1972). Osteology and relationships of the lanternfishes (family Myctophidae). Bull Nat Hist Mus LA.

[CR31] Posada D (2008). jModelTest: phylogenetic model averaging. Mol Biol Evol.

[CR32] Rambaut A, Drummond AJ (2007) Tracer v1.5, http://beast.bio.ed.ac.uk/Tracer

[CR33] Ray DL (1950). The peripheral nervous system of *Lampanyctus leucopsaurus*. J Morphol.

[CR34] Remane A (1971). Die grundlagen des naturlichen systems der vergleichenen anaotomie udn der phylogenetic.

[CR35] Rohlf FJ (2010a) tpsDIG. Department of Ecology and Evolution, State University of New York at Stony Brook. Version 2.16 http://life.bio.sunysb.edu/morph

[CR36] Rohlf FJ (2010b) tpsRelw. Department of Ecology and Evolution, State University of New York at Stony Brook. Version 1.49 http://life.bio.sunysb.edu/ morph

[CR37] Rohlf FJ, Slice DE (1990). Extensions of the procrustes method for the optimal superimposition of landmarks. Syst Zool.

[CR38] Rosen DE, Greenwood PH, Miles S, Patterson C (1973). Interrelationships of euteleostean fishes. Interrelationships of fishes.

[CR39] Sparks JS, Dunlap PV, Smith WL (2005). Evolution and diversification of a sexually dimorphic luminescent system in ponyfishes (Teleostei: Leiognathidae), including diagnoses for two new genera. Cladistics.

[CR40] Sutton TT, Hartel KE (2004). New species of Eustomias (Teleostei: Stomiidae) from the Western North Atlantic, with a revision of the subgenus Neostomias. Copeia.

[CR41] Sutton TT, Wiebe PH, Madin L, Bucklin A (2010). Diversity and community structure of pelagic fishes to 5,000 m depth in the Sargasso Sea. Deep-Sea Res II.

[CR42] Tåning AV (1918). Mediterranean Scopelidae (Saurus, Aulopus, Chlorophthalmus, and Myctophum). Rept Danish Ocean Exped 1908–1910.

[CR43] Turner JR, White EM, Collins MA, Partridge JC, Douglas RH (2009). Vision in lanternfish (Myctophidae): adaptations for viewing bioluminescence in the deep-sea. Deep-Sea Research I.

[CR44] Widder EA (2010). Bioluminescence in the ocean: origins of biological, chemical, and ecological diversity. Science.

