# Supplementary material for: Species-specific bioluminescence facilitates speciation in the deep sea
Source: Mar Biol. 2014 Feb 21;161(5):1139–48. doi: 10.1007/s00227-014-2406-x (PMC3996283; doi:10.1007/s00227-014-2406-x)
Supplement: Supplementary file 1 — Supplementary material 1 (PDF 8849 kb) [file 227_2014_2406_MOESM1_ESM.pdf]

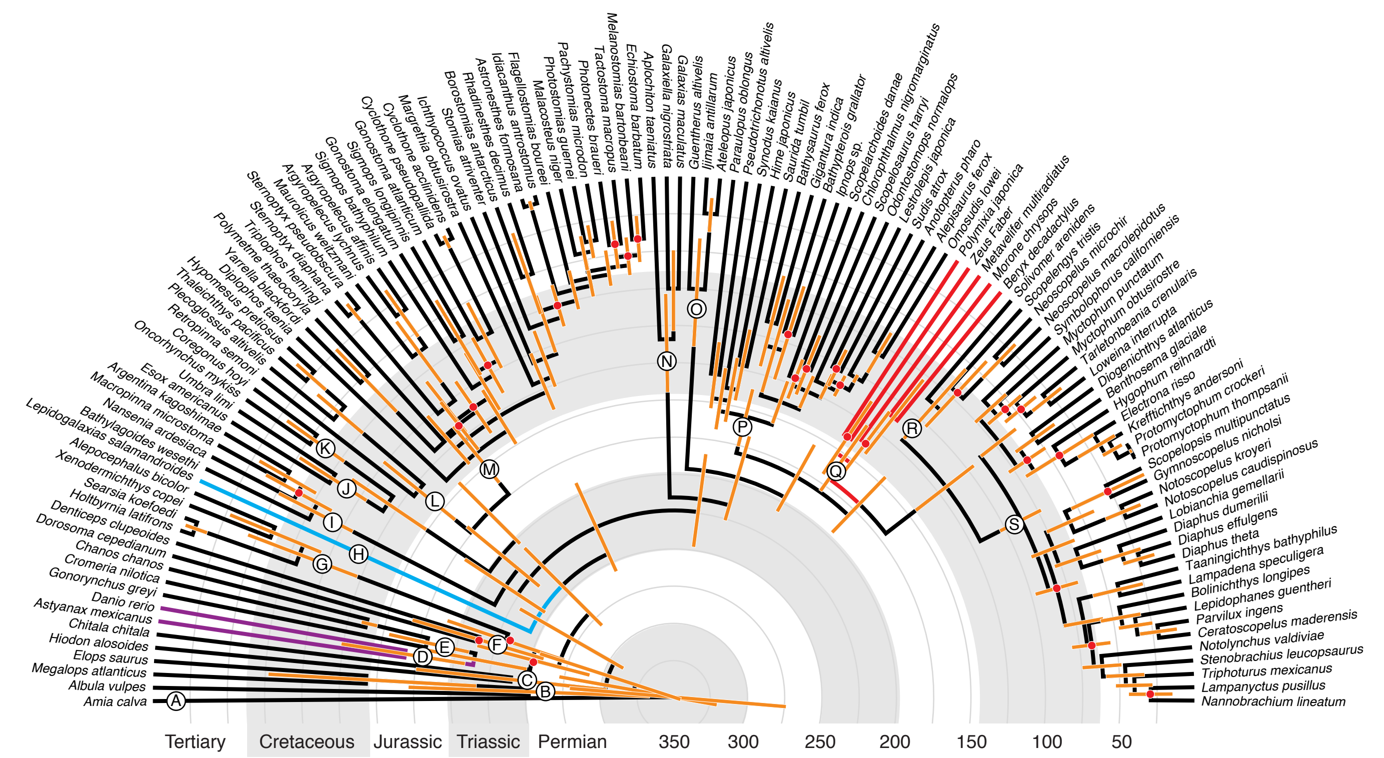

A Holostei (8 spp.)
B Elopomorpha (987 spp.)
C Osteoglossomorpha (230 spp.)
D Ostariophysi (9822 spp.)
E Gonorynchiformes (37 spp.)
F Clupeiformes (398 spp.)
G Alepocephaloids (137 spp.)
H Lepidogalaxiiformes (1 sp.)
I Argentiniformes (91 spp.)
J Esociformes (13 spp.)
K Salmoniformes (217 spp.)
L Osmeriformes (41 spp.)
M Stomiiformes (426 spp.)
N Galaxiidae (50 spp.)
O Ateleopodiformes (13 spp.)
P Aulopiformes (261 spp.)
Q Acanthomorpha (17946 spp.)
R Neoscopelidae (6 spp.)
S Myctophidae (252 spp.)

Estimated (MEDUSA) Multiple Rates
Background (Shown) r = 0.0143, ε = 0.926
Shift 1 (Increase, Acanthomorpha) r = 0.0516, ε = 0.647
Shift 2 (Increase, Ostariophysi) r = 0.0386, ε = 0.62
Shift 3 (Decrease, *Lepidogalaxias*) r = 0.008e-14, ε = 0.01
Posterior probability < 90%

Supplementary Figure 1. Temporal hypothesis of evolutionary relationships for ray-finned fishes with rate-shifts, 95% higher-posterior densities of estimated divergence times, and posterior probabilities at nodes indicated.

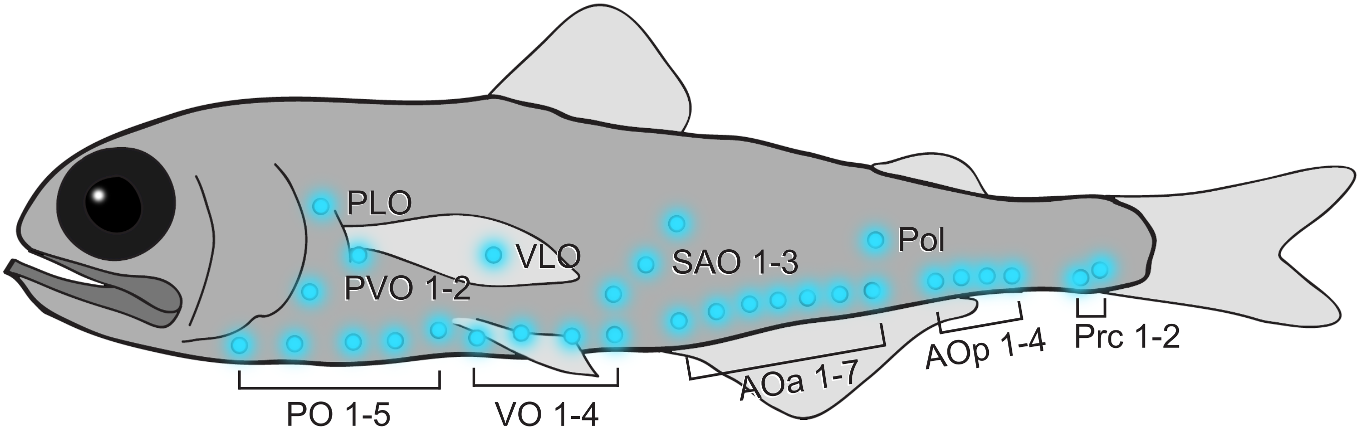

PLO - Suprapectoral organ series

PVO - Subpectoral organ series

VLO - Supraventral organ series

SAO - Supra-anal organs series

Pol - Postero-lateral organ series

Prc - Precaudal organs series

PO - Thoracic organs series

VO - Ventral organ series

AOa - Anterior anal organs series

AOp - Posterior anal organs series

Supplementary Figure 2. Illustration of a general lanternfish with bioluminescent photophores series found on the body labeled.

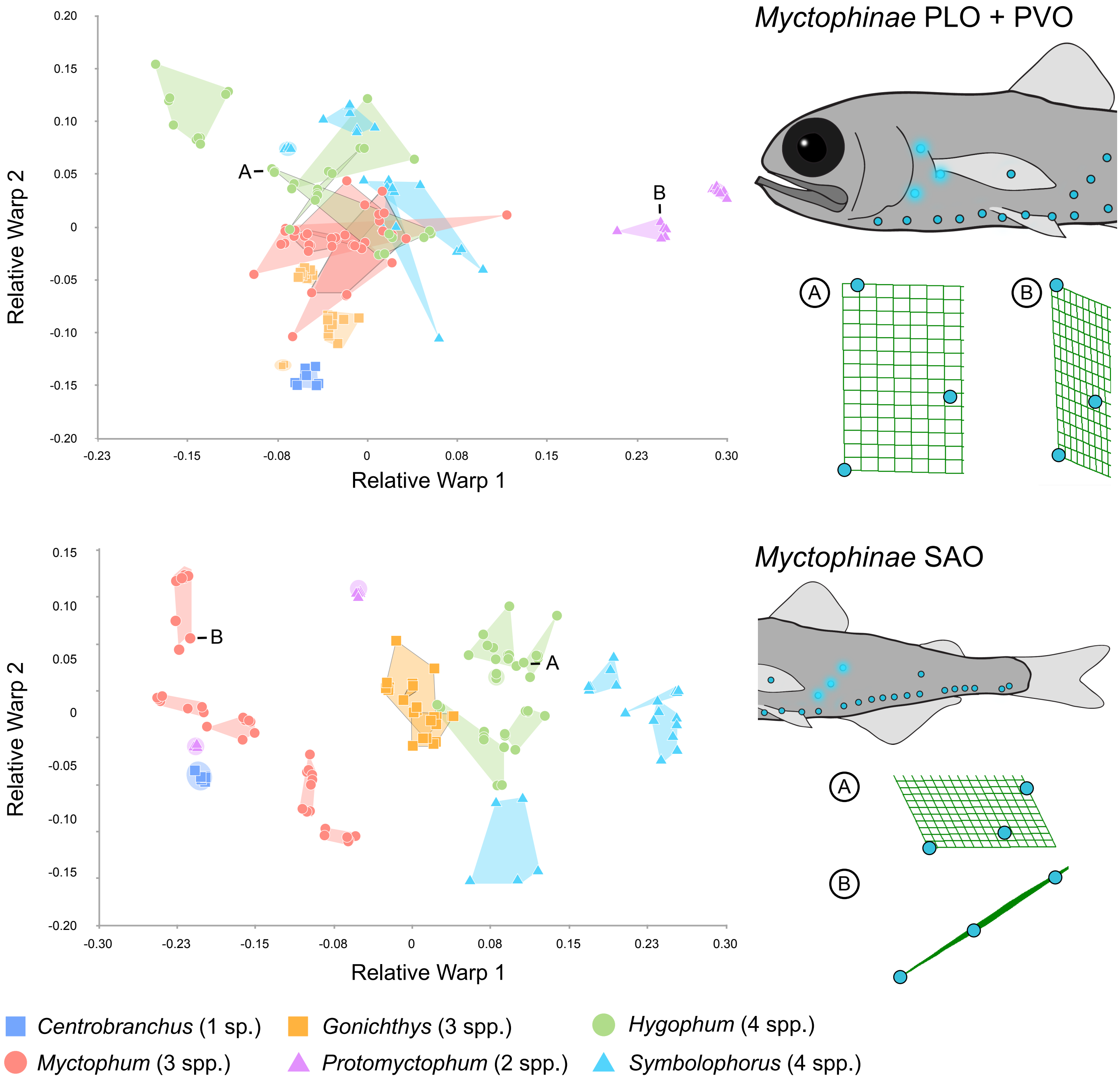

Supplementary Figure 3A. Relative warp analyses of lateral and ventral bioluminescent photophore series of lanternfishes investigated in this study. Includes analyses among genera in the subfamilies Myctophinae and Lampanyctinae, as well as analyses among species within genera.

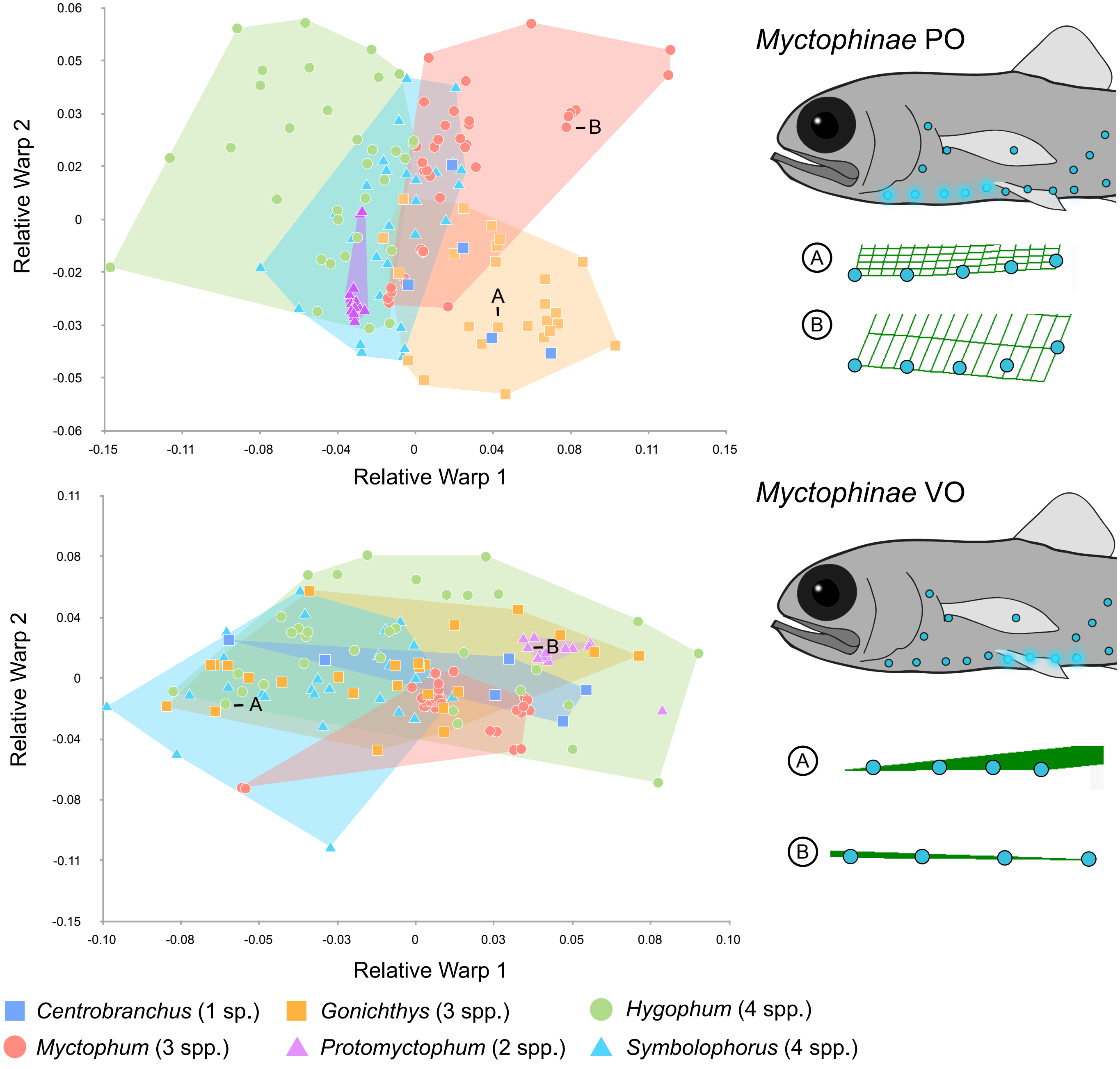

Supplementary Figure 3B. Relative warp analyses of lateral and ventral bioluminescent photophore series of lanternfishes investigated in this study. Includes analyses among genera in the subfamilies Myctophinae and Lampanyctinae, as well as analyses among species within genera.

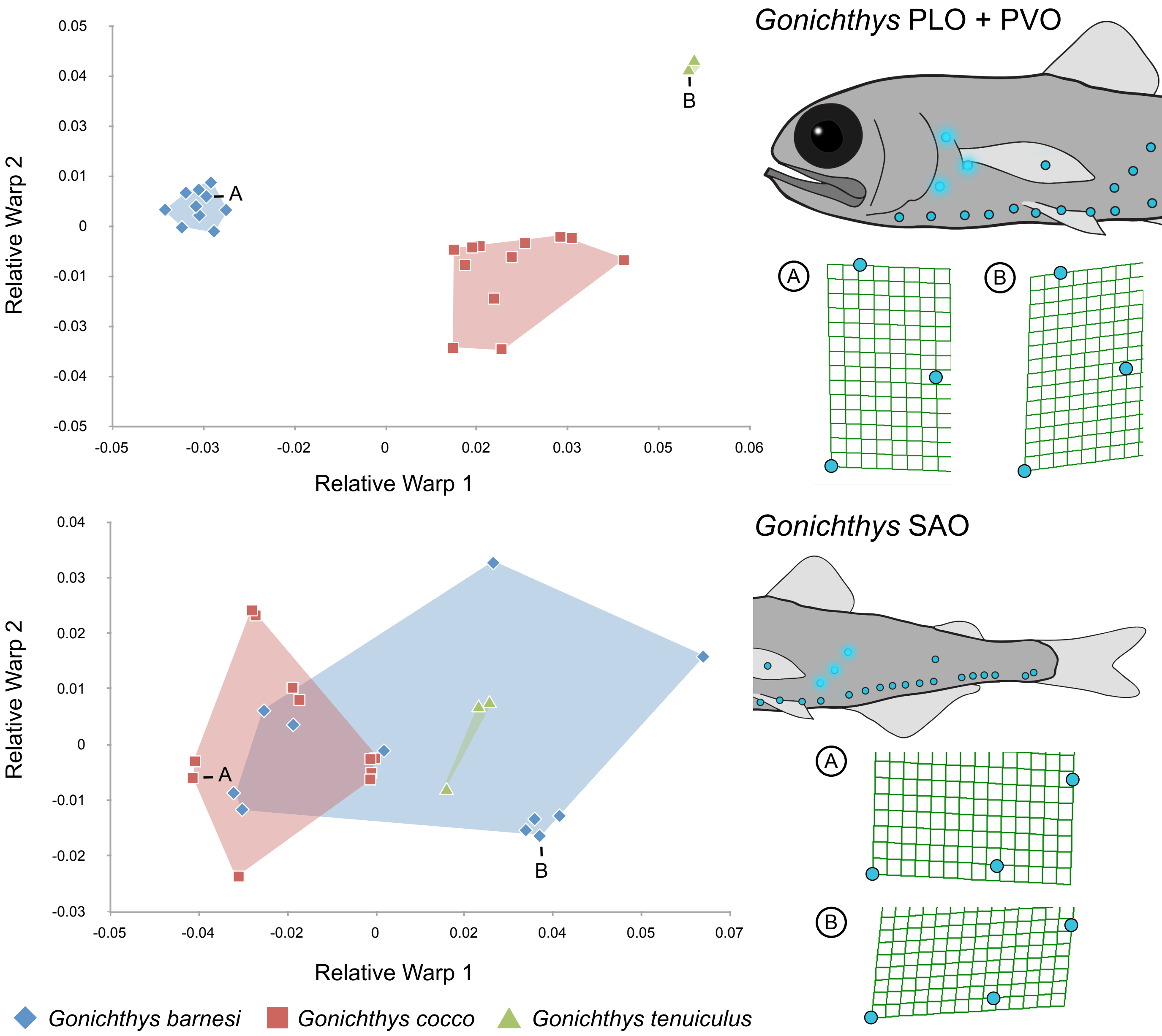

Supplementary Figure 3C. Relative warp analyses of lateral and ventral bioluminescent photophore series of lanternfishes investigated in this study. Includes analyses among genera in the subfamilies Myctophinae and Lampanyctinae, as well as analyses among species within genera.

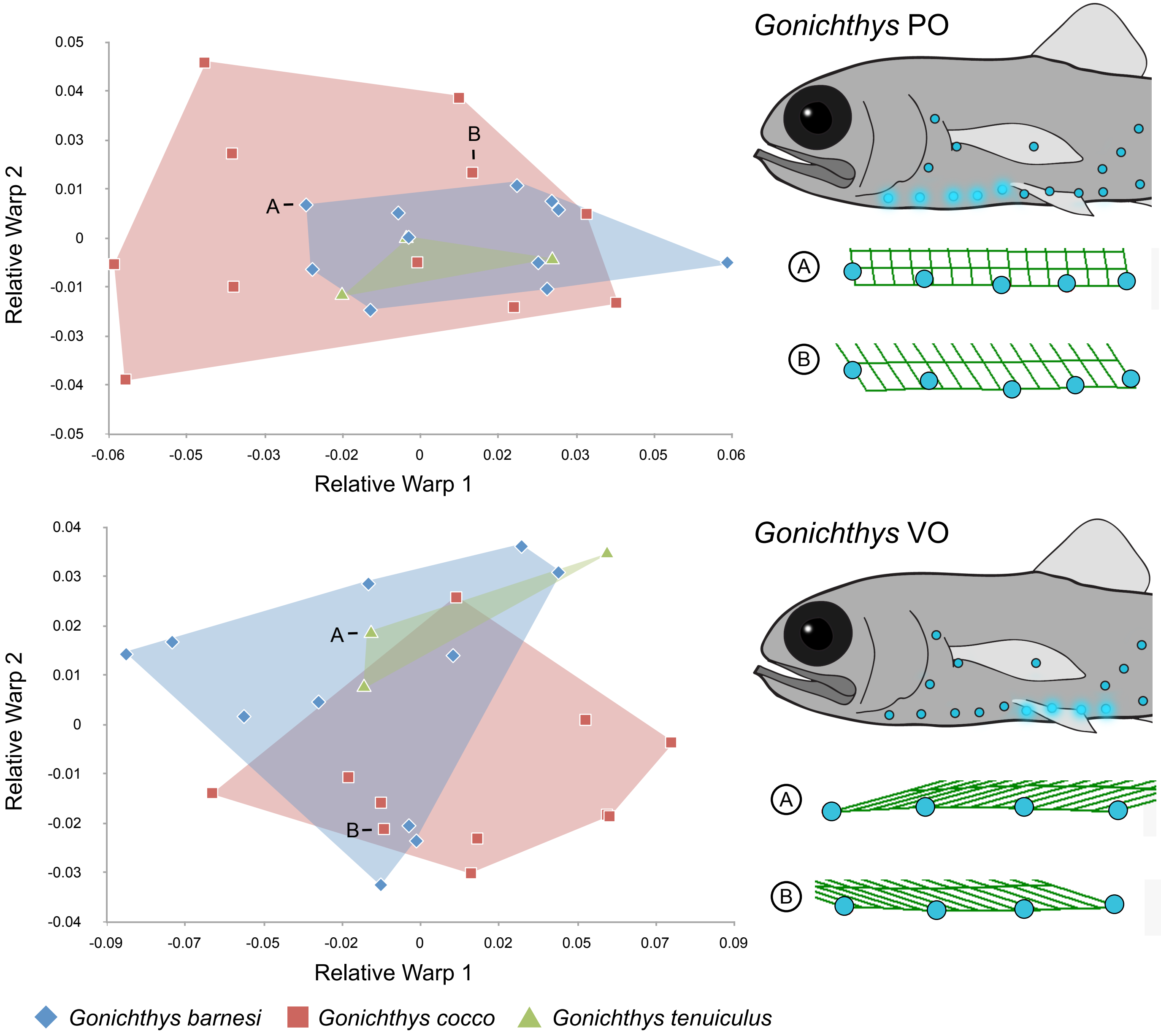

Supplementary Figure 3D. Relative warp analyses of lateral and ventral bioluminescent photophore series of lanternfishes investigated in this study. Includes analyses among genera in the subfamilies Myctophinae and Lampanyctinae, as well as analyses among species within genera.

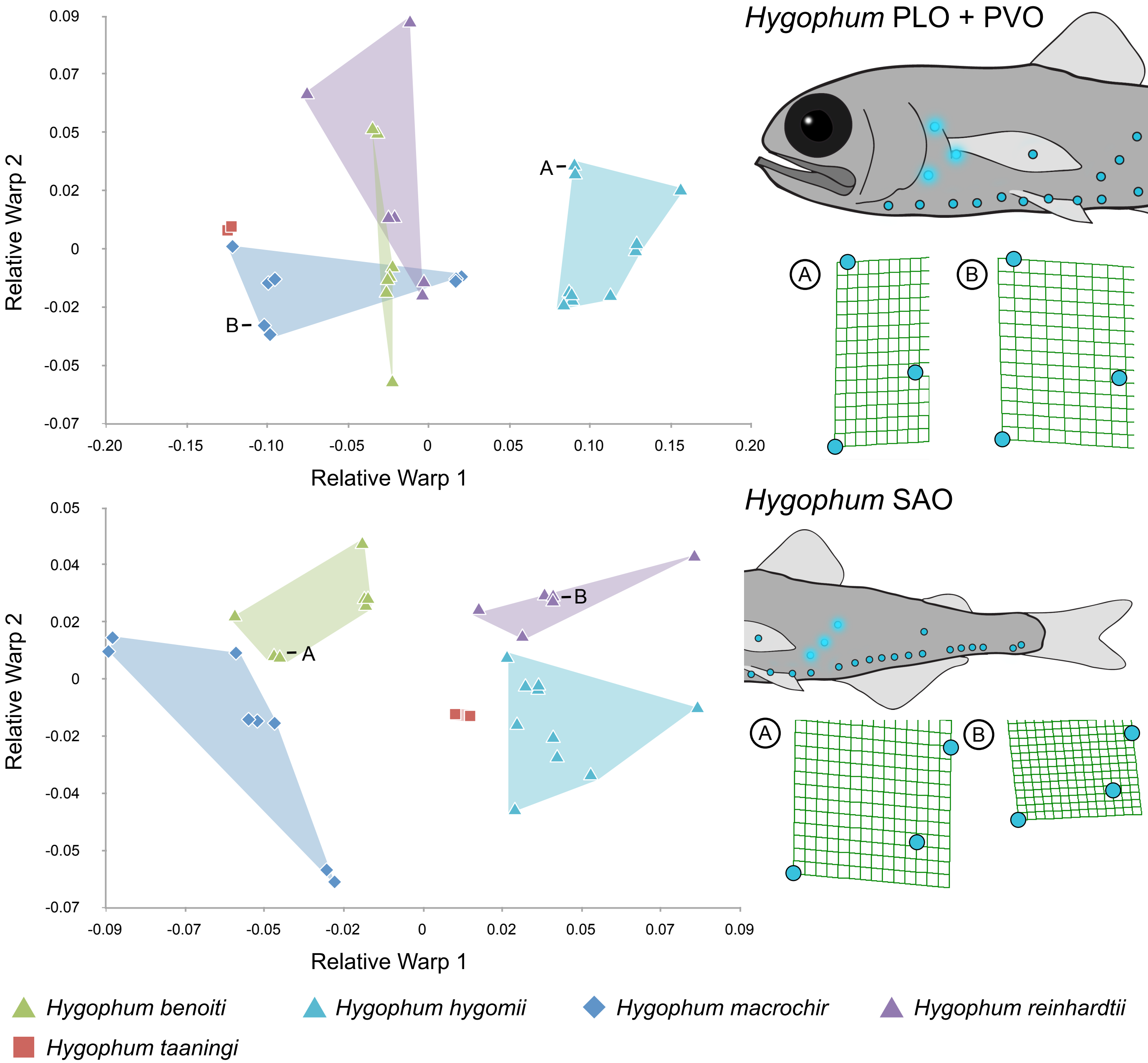

Supplementary Figure 3E. Relative warp analyses of lateral and ventral bioluminescent photophore series of lanternfishes investigated in this study. Includes analyses among genera in the subfamilies Myctophinae and Lampanyctinae, as well as analyses among species within genera.

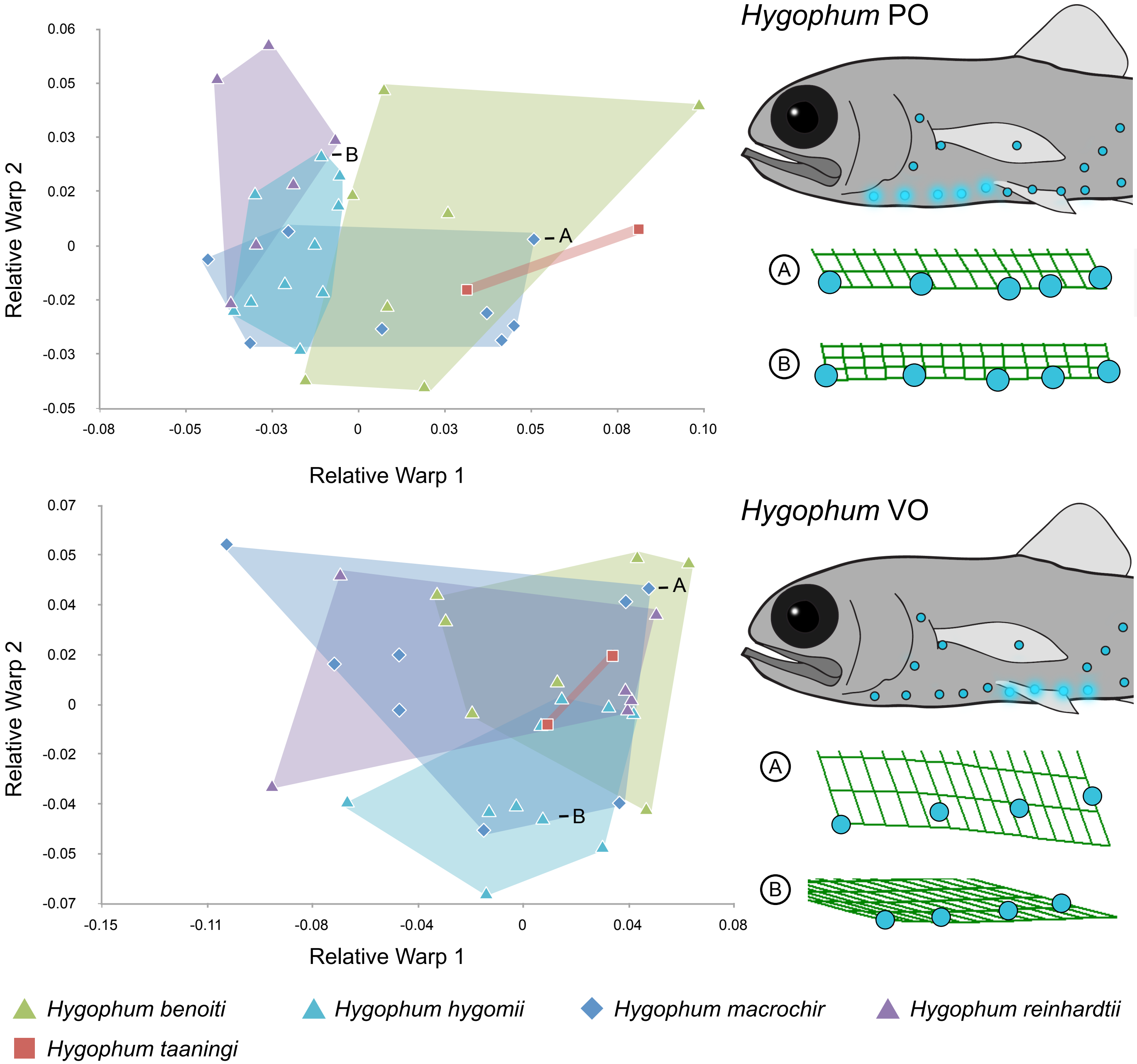

Supplementary Figure 3F. Relative warp analyses of lateral and ventral bioluminescent photophore series of lanternfishes investigated in this study. Includes analyses among genera in the subfamilies Myctophinae and Lampanyctinae, as well as analyses among species within genera.

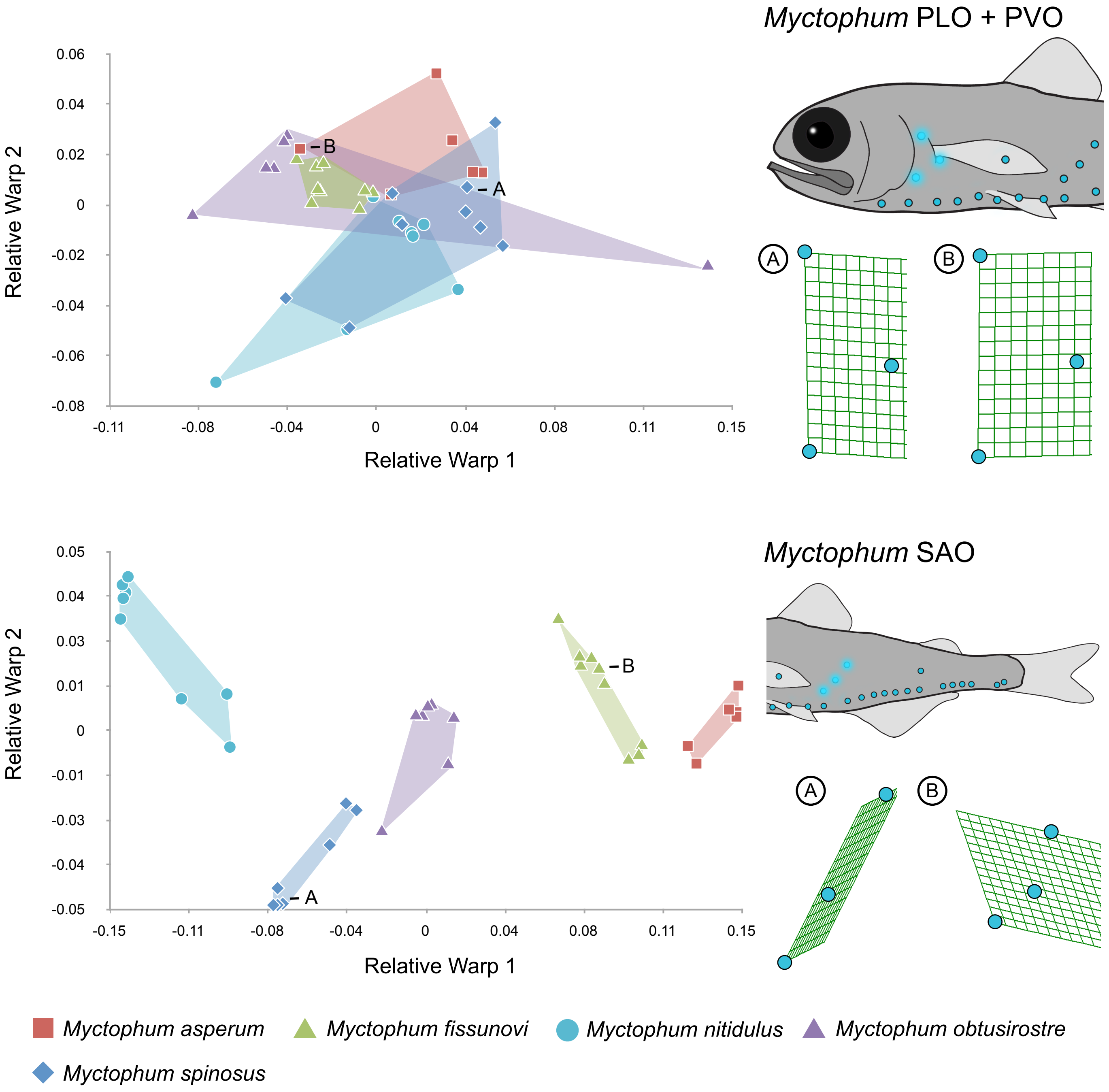

Supplementary Figure 3G. Relative warp analyses of lateral and ventral bioluminescent photophore series of lanternfishes investigated in this study. Includes analyses among genera in the subfamilies Myctophinae and Lampanyctinae, as well as analyses among species within genera.

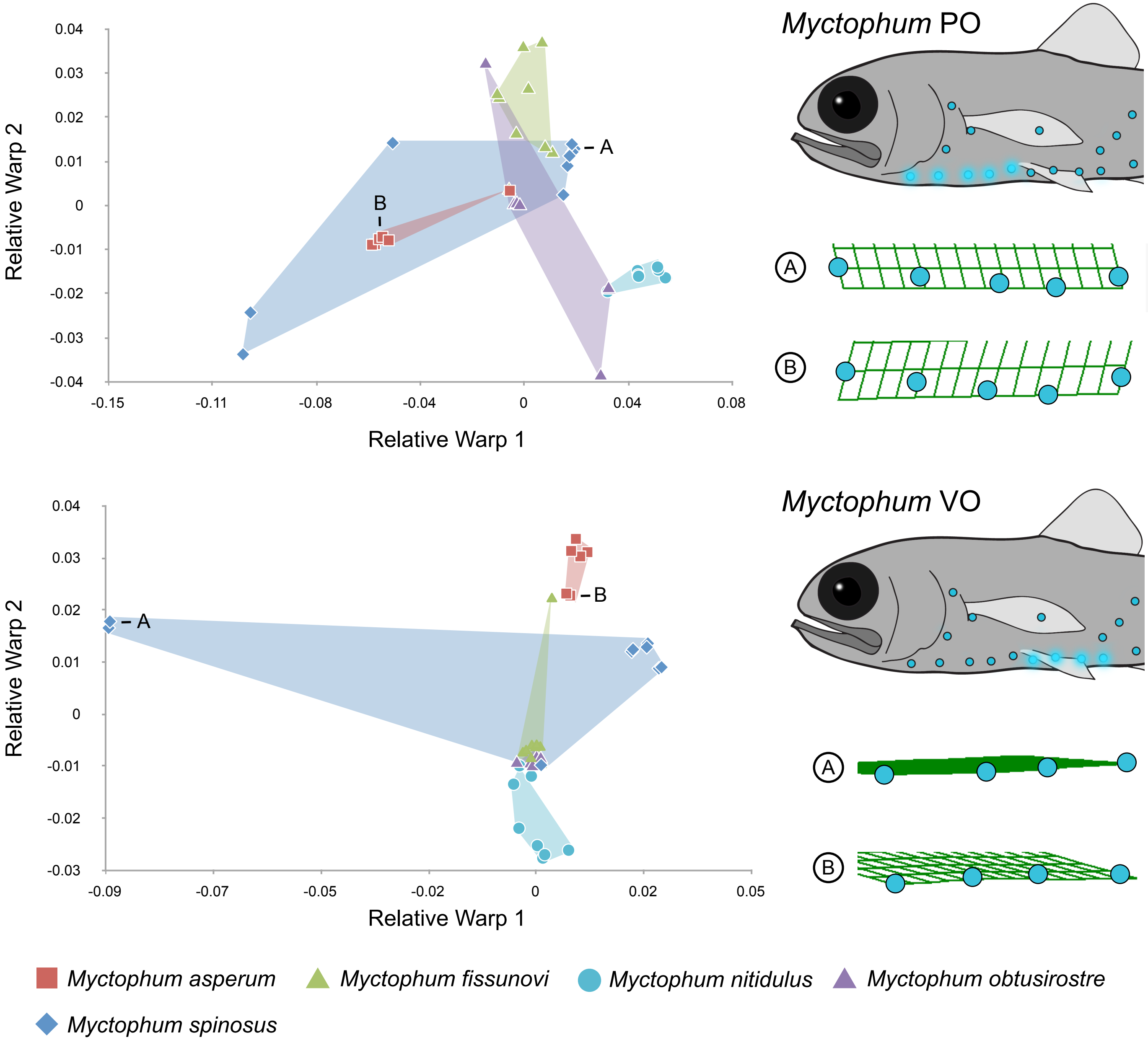

Supplementary Figure 3H. Relative warp analyses of lateral and ventral bioluminescent photophore series of lanternfishes investigated in this study. Includes analyses among genera in the subfamilies Myctophinae and Lampanyctinae, as well as analyses among species within genera.

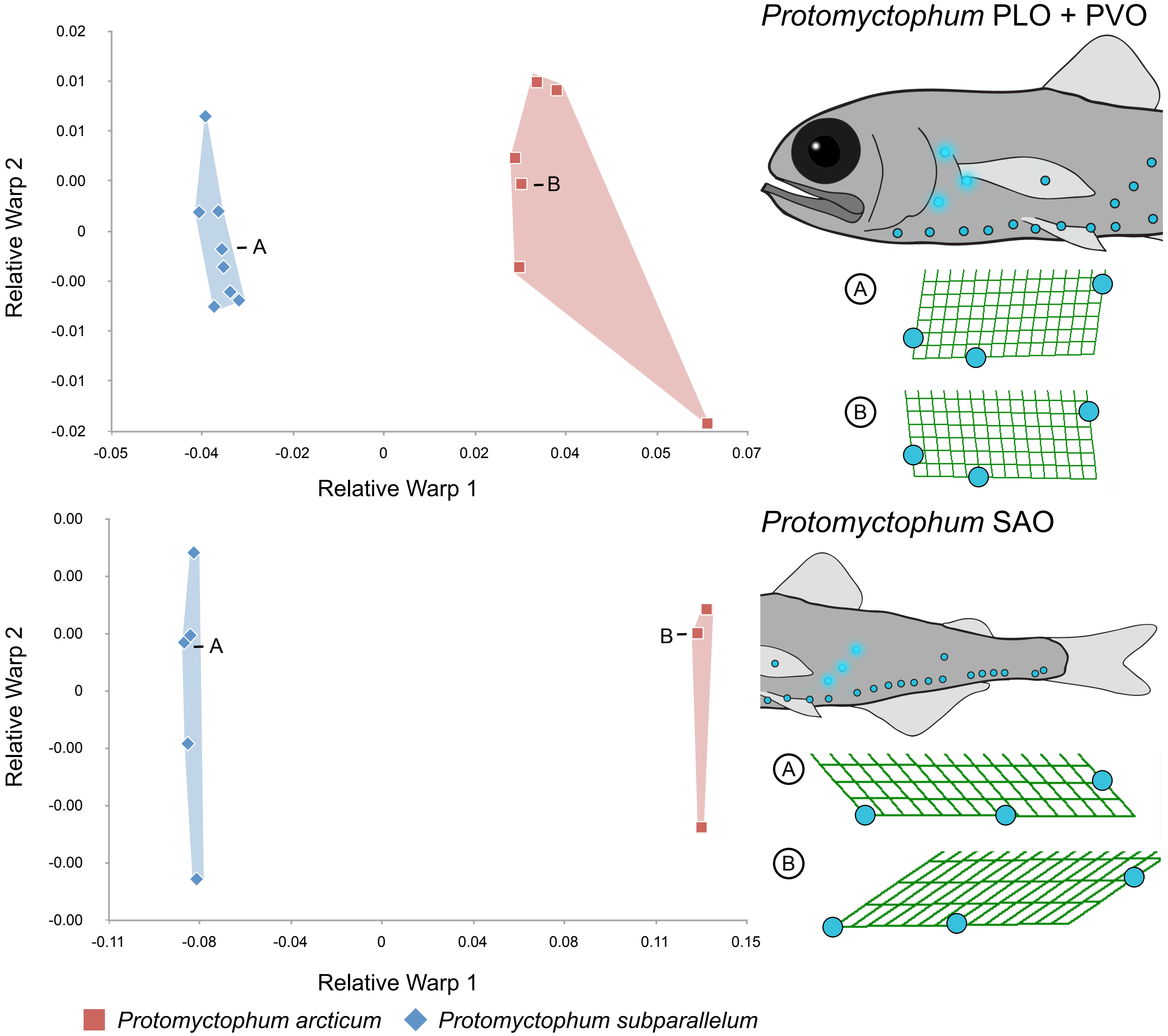

Supplementary Figure 3I. Relative warp analyses of lateral and ventral bioluminescent photophore series of lanternfishes investigated in this study. Includes analyses among genera in the subfamilies Myctophinae and Lampanyctinae, as well as analyses among species within genera.

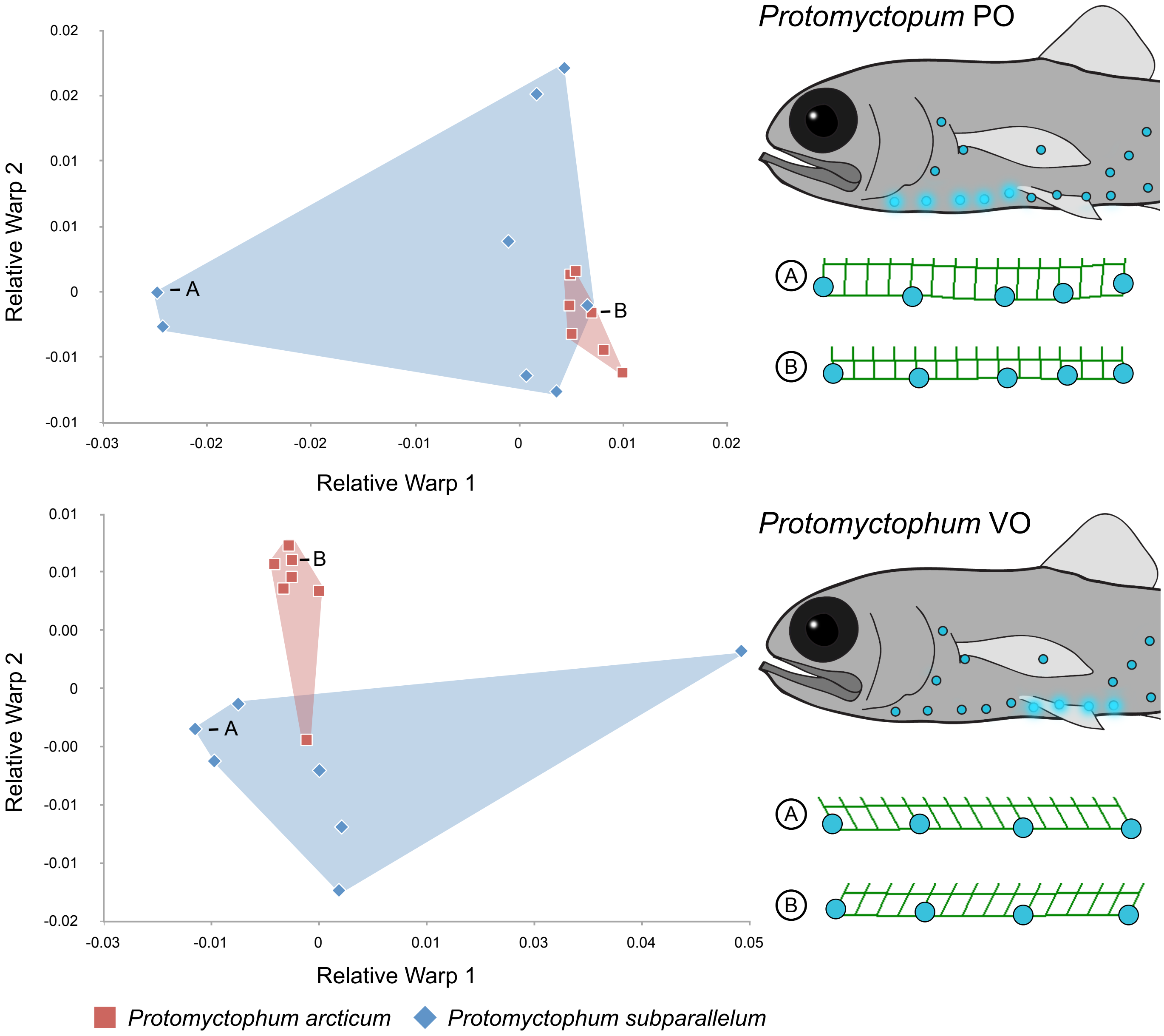

Supplementary Figure 3J. Relative warp analyses of lateral and ventral bioluminescent photophore series of lanternfishes investigated in this study. Includes analyses among genera in the subfamilies Myctophinae and Lampanyctinae, as well as analyses among species within genera.

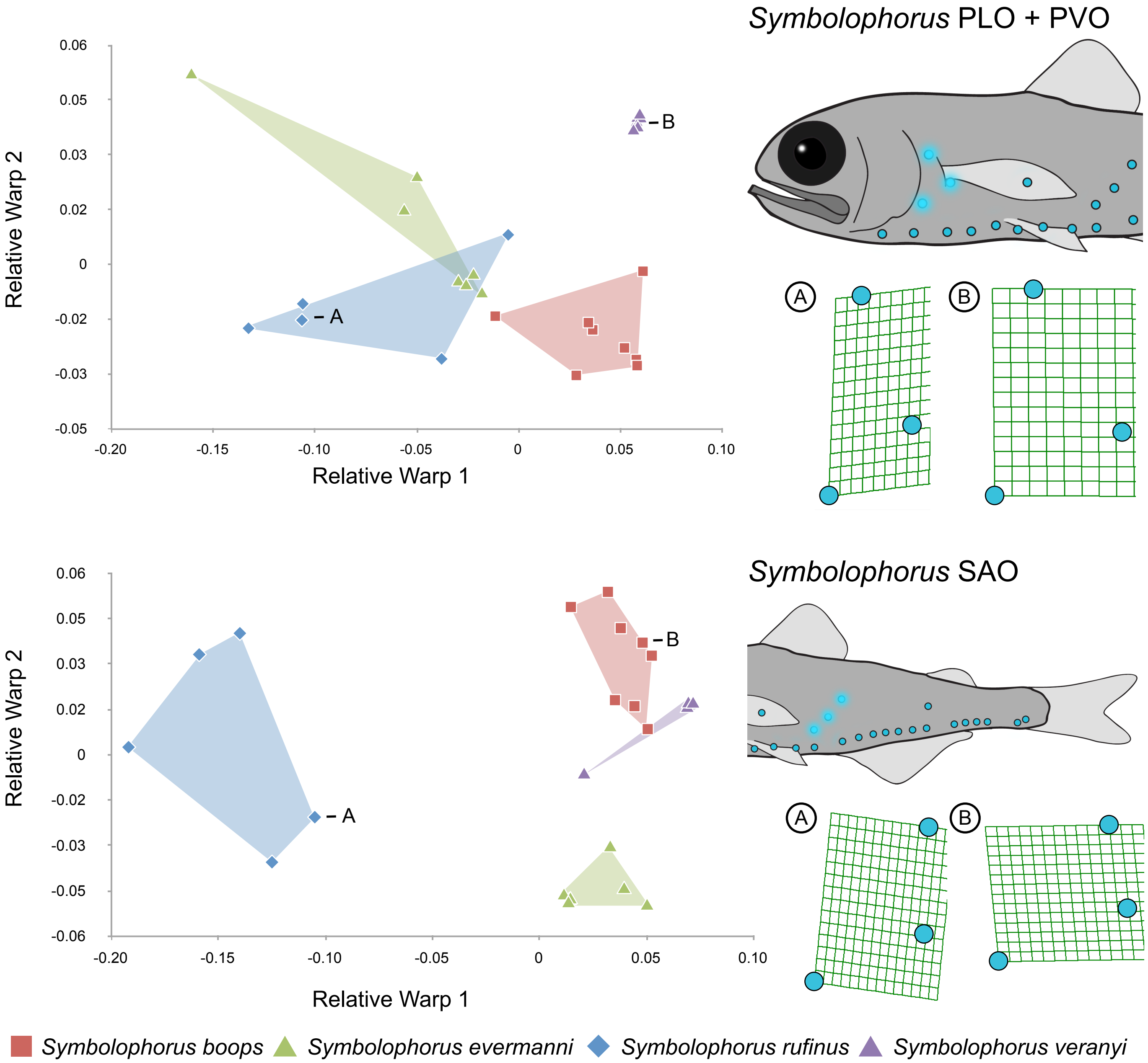

Supplementary Figure 3K. Relative warp analyses of lateral and ventral bioluminescent photophore series of lanternfishes investigated in this study. Includes analyses among genera in the subfamilies Myctophinae and Lampanyctinae, as well as analyses among species within genera.

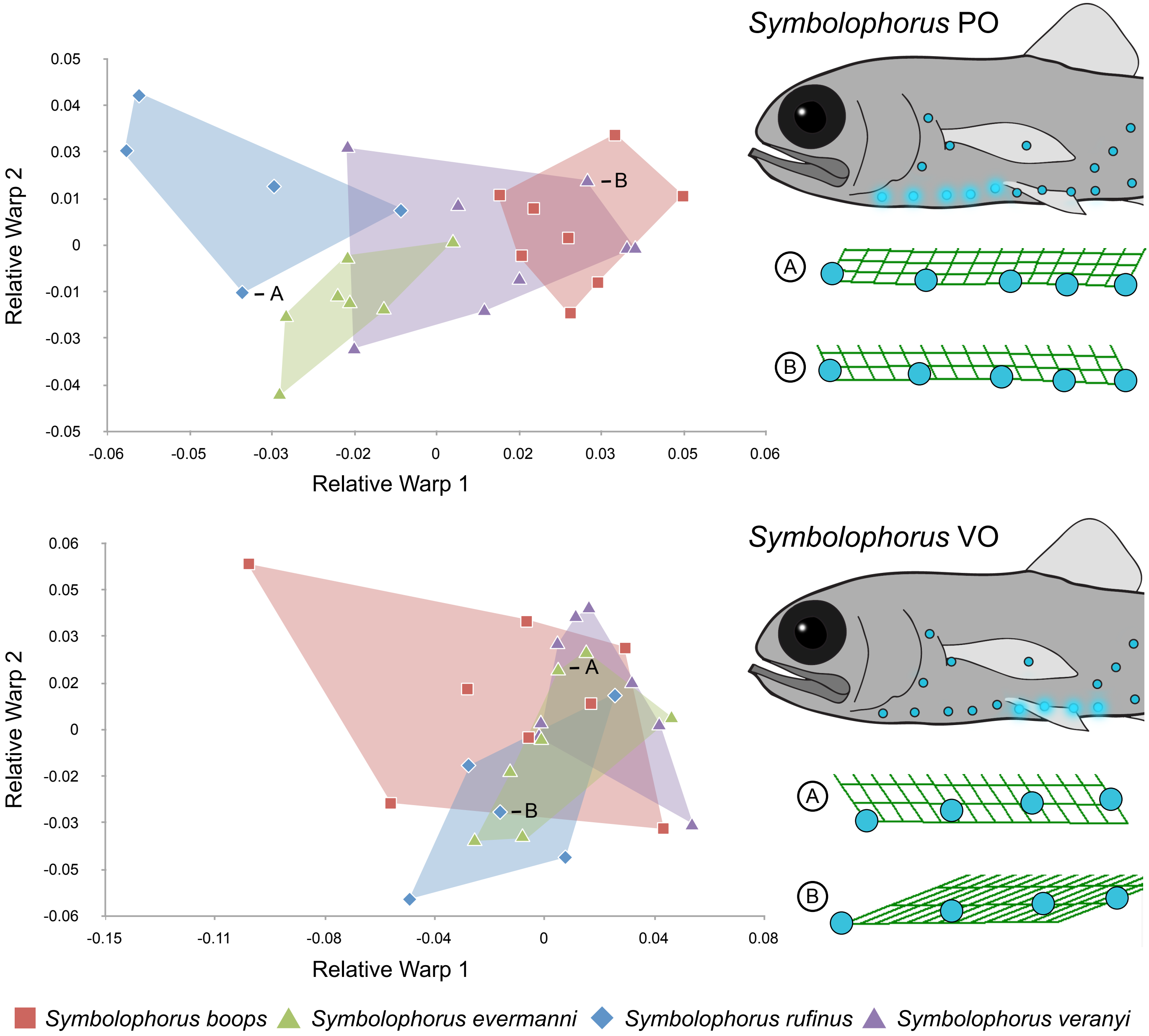

Supplementary Figure 3L. Relative warp analyses of lateral and ventral bioluminescent photophore series of lanternfishes investigated in this study. Includes analyses among genera in the subfamilies Myctophinae and Lampanyctinae, as well as analyses among species within genera.

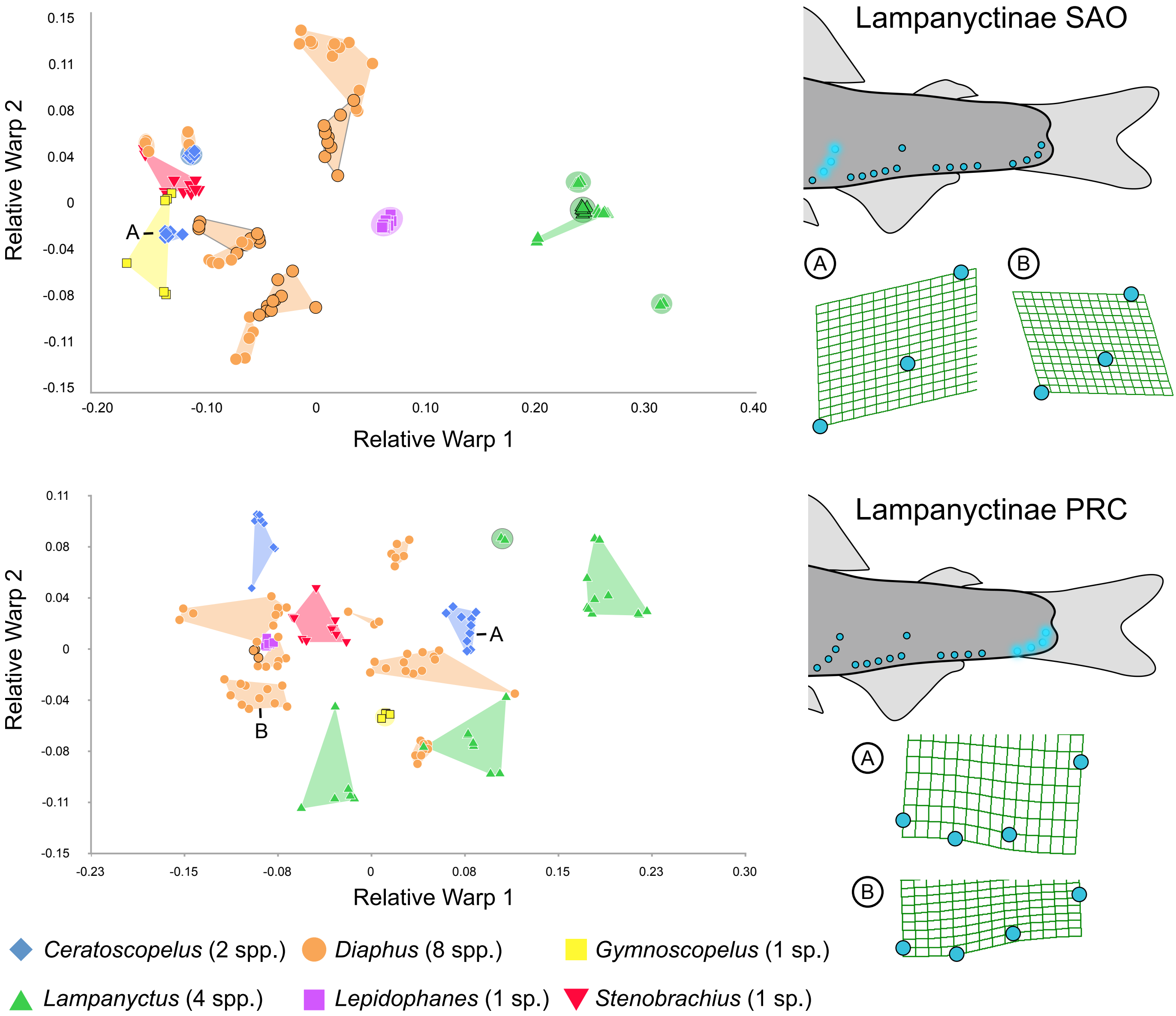

Supplementary Figure 3M. Relative warp analyses of lateral and ventral bioluminescent photophore series of lanternfishes investigated in this study. Includes analyses among genera in the subfamilies Myctophinae and Lampanyctinae, as well as analyses among species within genera.

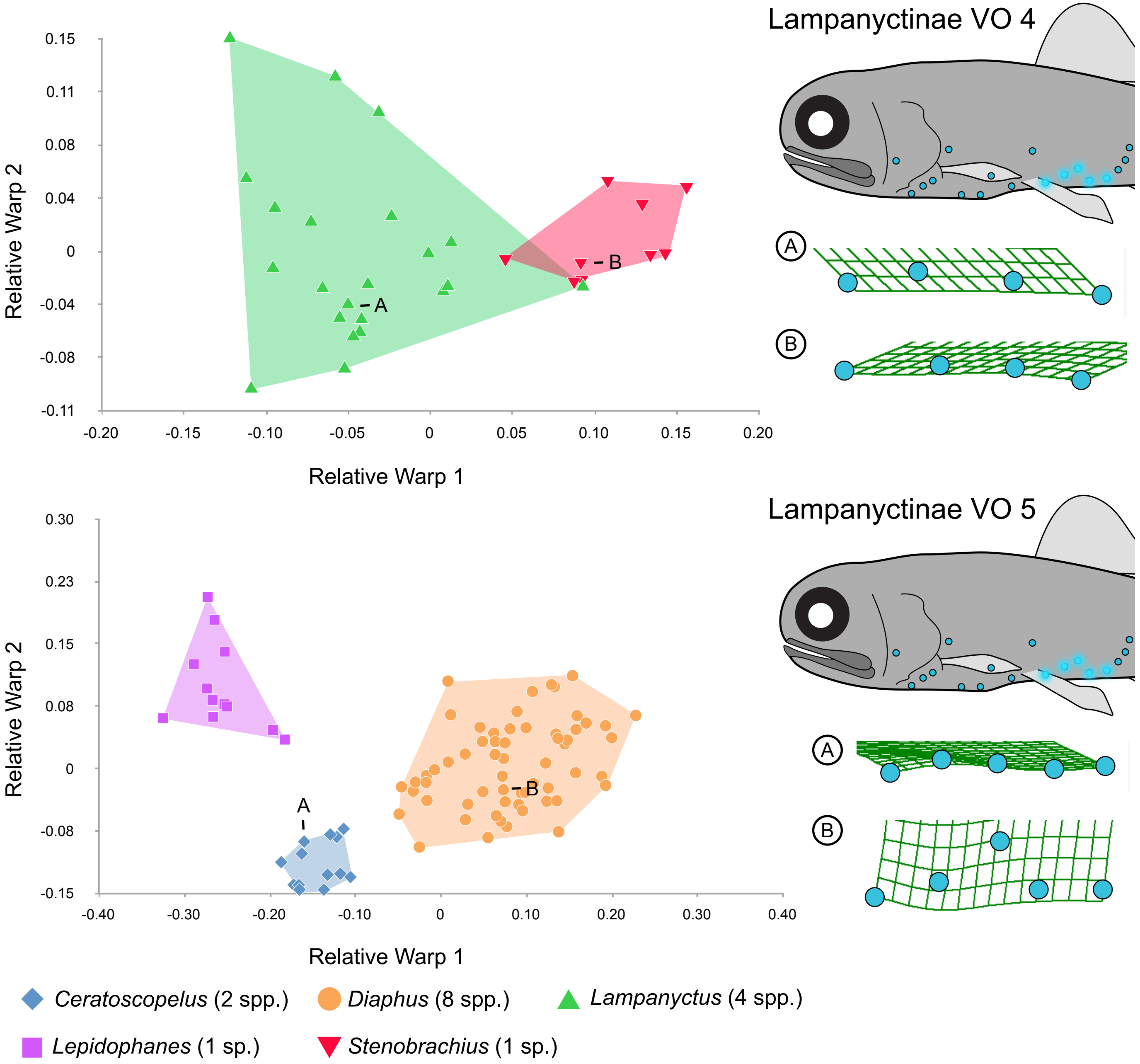

Supplementary Figure 3N. Relative warp analyses of lateral and ventral bioluminescent photophore series of lanternfishes investigated in this study. Includes analyses among genera in the subfamilies Myctophinae and Lampanyctinae, as well as analyses among species within genera.

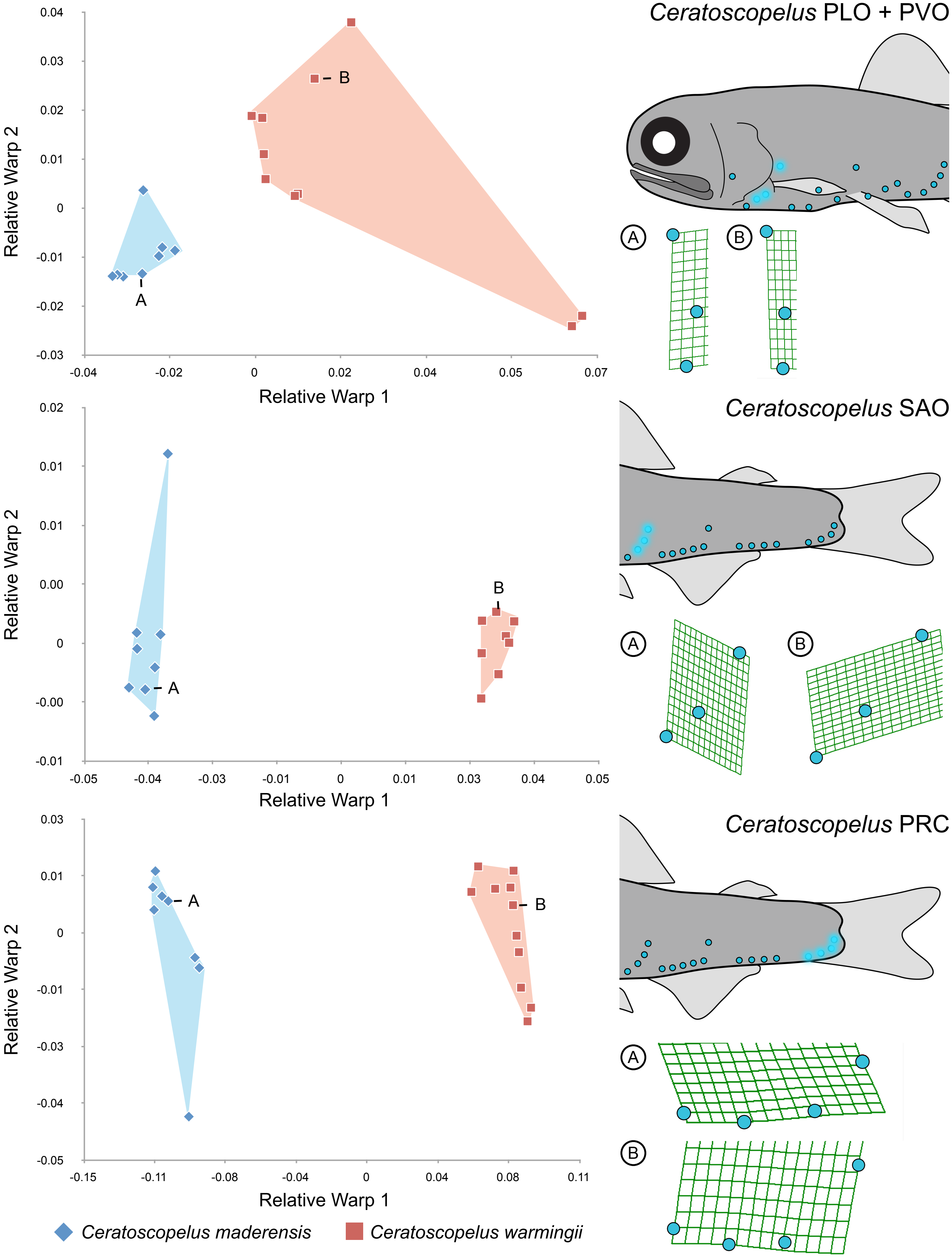

Supplementary Figure 3O. Relative warp analyses of lateral and ventral bioluminescent photophore series of lanternfishes investigated in this study. Includes analyses among genera in the subfamilies Myctophinae and Lampanyctinae, as well as analyses among species within genera.

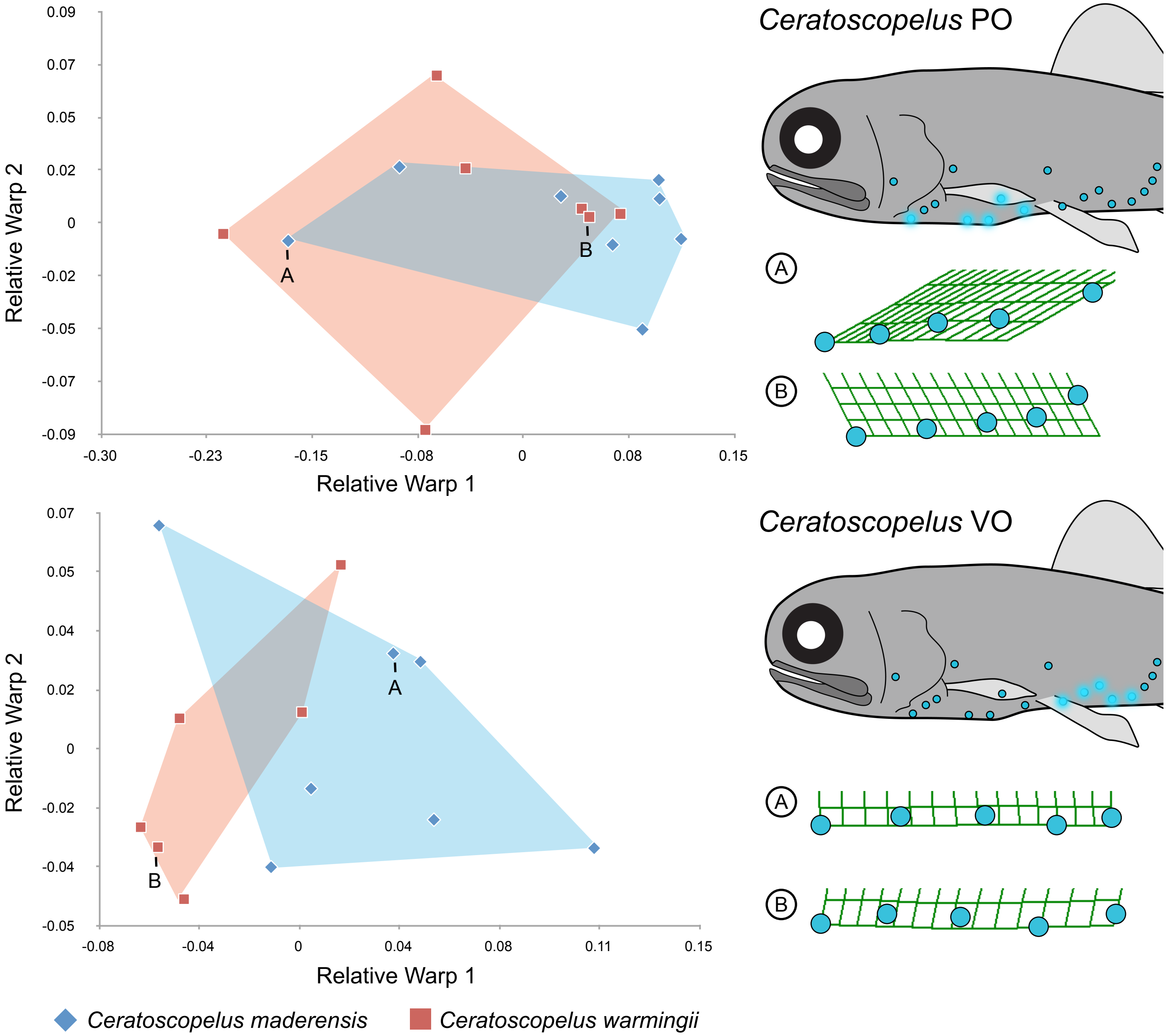

Supplementary Figure 3P. Relative warp analyses of lateral and ventral bioluminescent photophore series of lanternfishes investigated in this study. Includes analyses among genera in the subfamilies Myctophinae and Lampanyctinae, as well as analyses among species within genera.

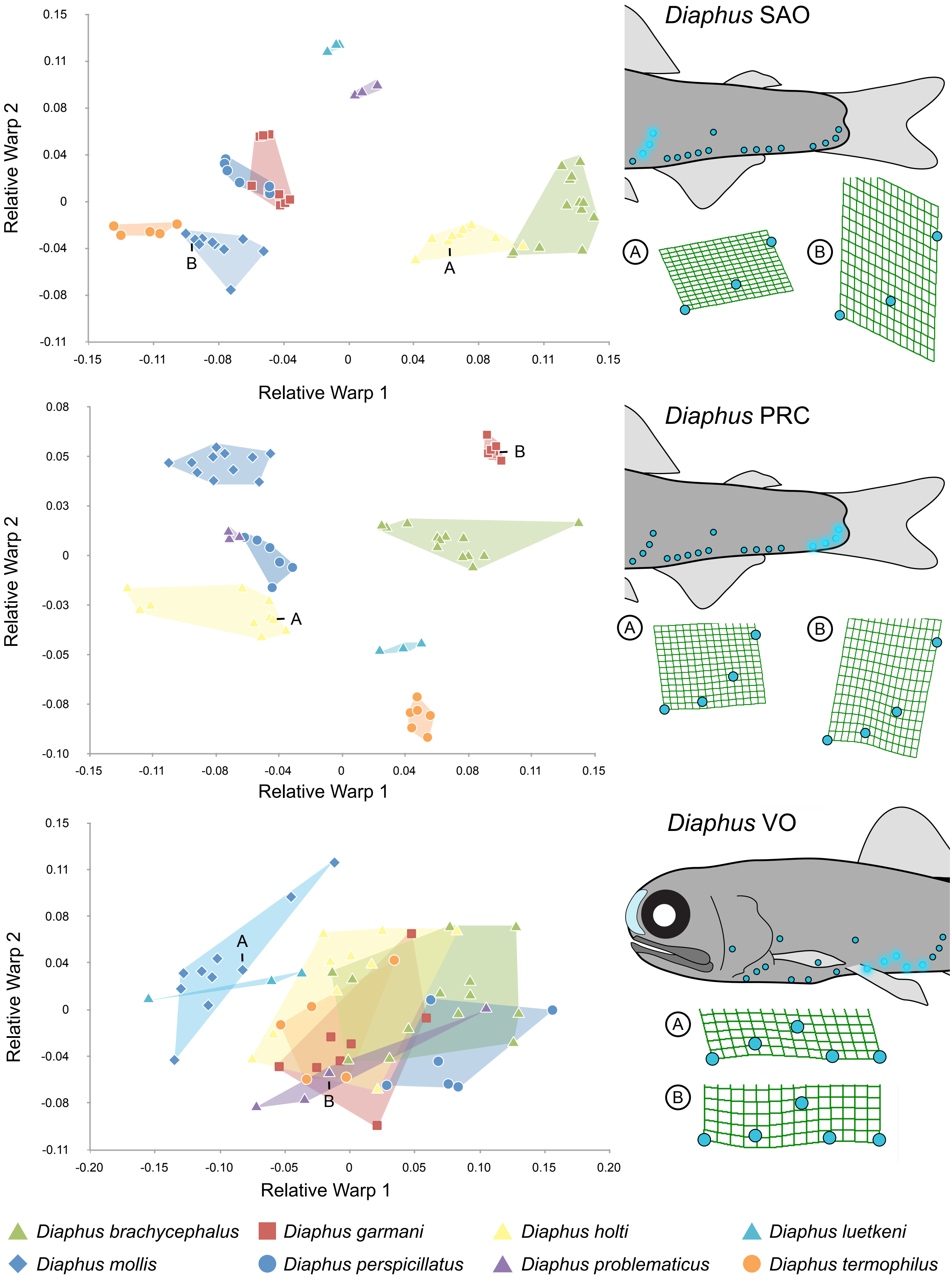

Supplementary Figure 3Q. Relative warp analyses of lateral and ventral bioluminescent photophore series of lanternfishes investigated in this study. Includes analyses among genera in the subfamilies Myctophinae and Lampanyctinae, as well as analyses among species within genera.

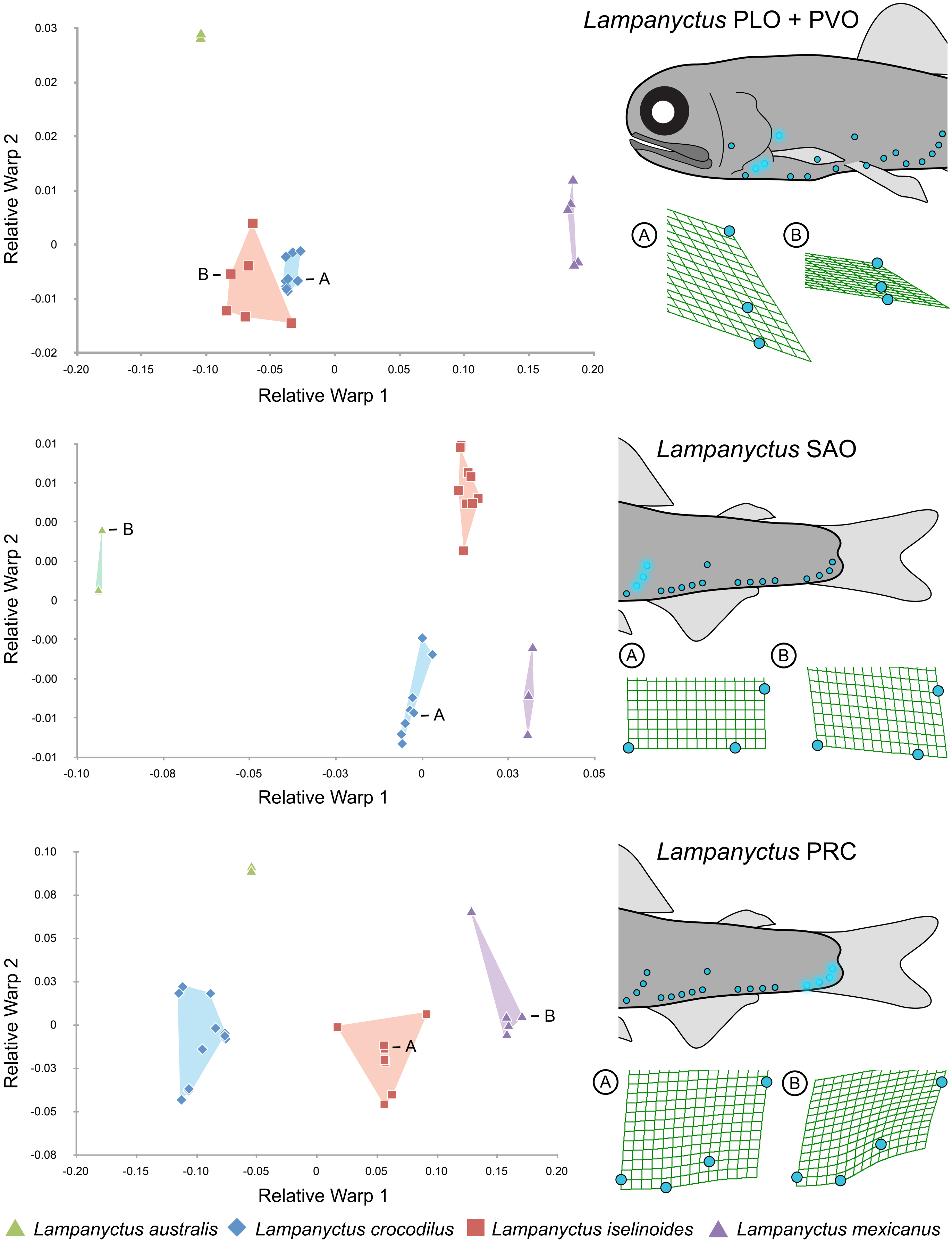

Supplementary Figure 3R. Relative warp analyses of lateral and ventral bioluminescent photophore series of lanternfishes investigated in this study. Includes analyses among genera in the subfamilies Myctophinae and Lampanyctinae, as well as analyses among species within genera.

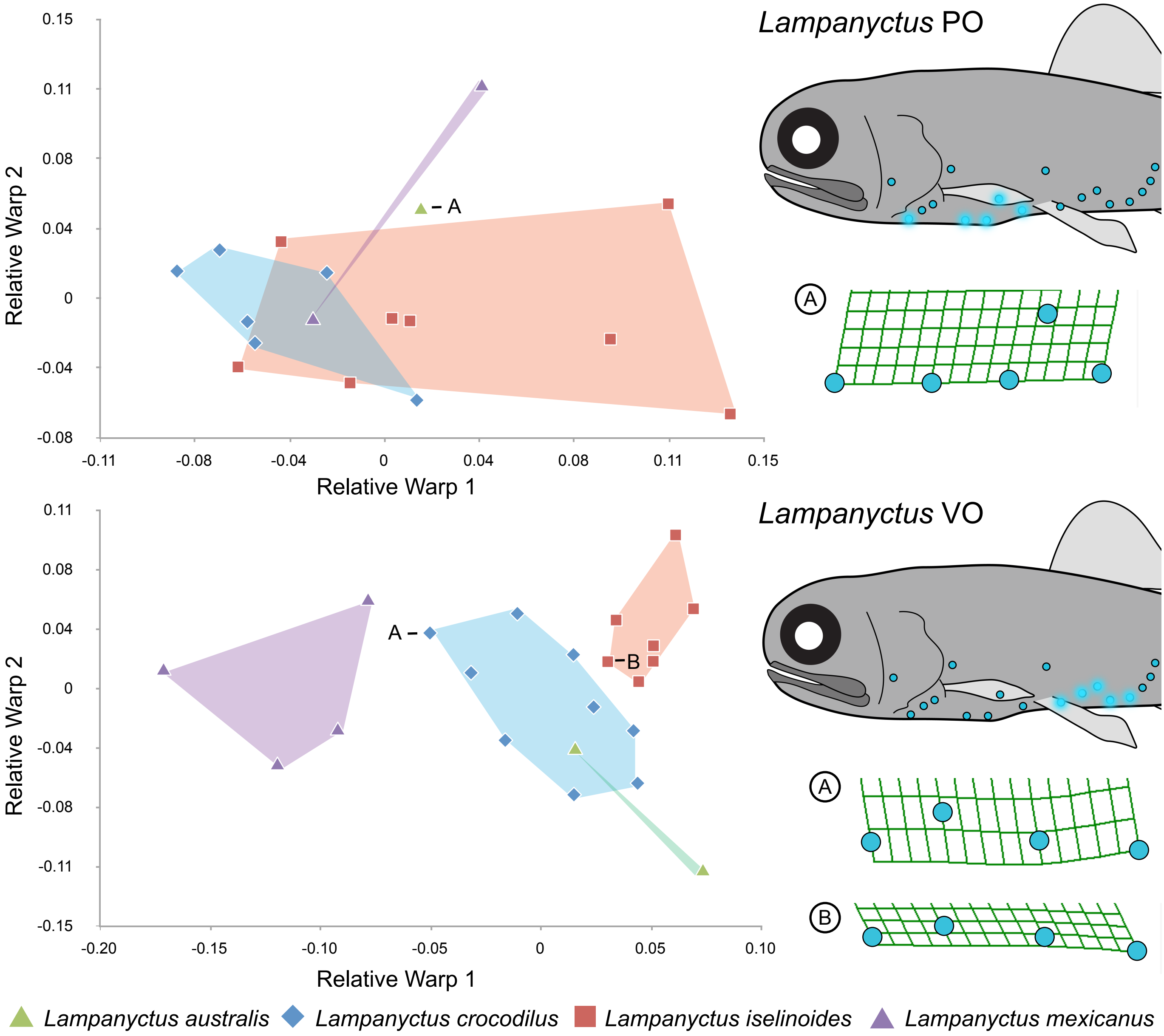

Supplementary Figure 3S. Relative warp analyses of lateral and ventral bioluminescent photophore series of lanternfishes investigated in this study. Includes analyses among genera in the subfamilies Myctophinae and Lampanyctinae, as well as analyses among species within genera.

Supplementary Table 1: Taxonomic sampling and GenBank accession numbers for phylogenetic analyses.

Family	Species	Voucher for New Sequences	RAG1	ZIC1	COI
**Amiidae**	*Amia calva*		AY430199	EF032909	AB042952
**Albulidae**	*Albula vulpes*		JX190803	JX191247	GU225131
**Elopidae**	*Elops saurus*		FJ896408	JX191250	AP004807
**Megalopidae**	*Megalops atlanticus*		AY430204	JX191251	AP004808.
**Hiodontidae**	*Hiodon alosoides*		AY430200	EU366766	AP004356
**Notopteridae**	*Chitala chitala*		FJ896406	JX191260	FJ918897
**Alepocephalidae**	*Alepocephalus bicolor*	FMNH 120786	Unavailable	KJ190108	KJ190022
**Alepocephalidae**	*Xenodermichthys copei*		FJ896433	FJ906652	FJ918920
**Platytroctidae**	*Holtbyrnia latifrons*	FMNH Uncat Field RGS7T3	Unavailable	KJ190109	KJ190023
**Platytroctidae**	*Searsia koefoedi*		Unavailable	JX191274	EU148320
**Denticipitidae**	*Denticeps clupeoides*		DQ912100	Unavailable	AP007276
**Clupeidae**	*Dorosoma cepdianum*		DQ912099	EU366767	EU366583
**Chanidae**	*Chanos chanos*		JX190809	JX191264	HQ654700
**Gonorynchidae**	*Gonorhynchus greyi*		JX190810	JX191265	AB054134
**Kneriidae**	*Cromeria nilotica*		JX190811	JX191266	AP007275
**Cyprinidae**	*Danio rerio*		U71093	EF032910	NC002333
**Characidae**	*Astyanax mexicanus*		HQ289318	Unavailable	HQ557152
**Lepidogalaxiidae**	*Lepidogalaxias salamanderi*		FJ896438	JX191288	FJ918925
**Plecoglossidae**	*Plecoglossus altivelis*	FMNH 120788	KJ190076	KJ190111	KJ190025
**Bathylagidae**	*Bathylagoides wesethi*	FMNH 122151	KJ190075	KJ190110	KJ190024
**Microstomatidae**	*Nansenia ardesiaca*		Unavailable	JX191277	AP004106
**Opisthoproctidae**	*Macropinna microstoma*		JX190823	JX191278	FJ164828
**Argentinidae**	*Argentina kagoshimae*	FMNH 120790	KJ190078	KJ190113	KJ190027
**Esocidae**	*Esox americanus*		JX190824	JX191279	EU524569
**Umbridae**	*Umbra limi*		JX190826	JX191281	EU522452
**Salmonidae**	*Oncorhynchus mykiss*		EF032911	EF032976	HQ167682
**Salmonidae**	*Coregonus hoyi*	FMNH 117754	KJ190077	KJ190112	KJ190026
**Retropinnidae**	*Retropinna semoni*		JX190829	JX191284	FJ918924
**Osmeridae**	*Thaleichthys pacificus*		AY380537	EU366774	FJ165413
**Osmeridae**	*Hypomesus pretiosus*		JX190828	JX191283	JQ354137
**Diplophidae**	*Diplophos taenia*	FMNH 120801	KJ190079	KJ190114	KJ190028
**Diplophidae**	*Triplophos hemingi*	FMNH 120799	KJ190084	KJ190123	KJ190038
**Phosichthyidae**	*Ichthyococcus ovatus*		GQ860317	Unavailable	GQ860370
**Phosichthyidae**	*Polymetme thaeocoryla*	MCZ 158591/KUIT 3547	KJ190085	KJ190124	KJ190039
**Phosichthyidae**	*Yarrella blackfordi*		FJ896448	FJ906669	FJ918935
**Sternoptychidae**	*Argyropelecus affinis*	FMNH 122177	Unavailable	KJ190121	KJ190035
**Sternoptychidae**	*Argyropelecus lychnus*	FMNH Uncat Field RGS6	KJ190082	KJ190120	KJ190034
**Sternoptychidae**	*Maurolicus weitzmani*	KUI 28081/KUIT 2975	GQ860309	Unavailable	KJ190037
**Sternoptychidae**	*Sternoptyx diaphana*	SIO 09-325	KJ190083	KJ190122	KJ190036
**Sternoptychidae**	*Sternoptyx pseudobscura*		KF768159	KF768165	KF768176
**Gonostomatidae**	*Cyclothone acclinidens*		GQ860304	Unavailable	GQ860355
**Gonostomatidae**	*Cyclothone pseudopallida*		GQ860307	Unavailable	GQ860358
**Gonostomatidae**	*Gonostoma atlanticum*	MCZ 157341/KUIT 3114	Unavailable	KJ190117	KJ190031
**Gonostomatidae**	*Gonostoma elongatum*	MCZ 158779/KUIT 3664	KJ190081	KJ190116	KJ190030
**Gonostomatidae**	*Margrethia obtusirostra*	KUIT 3305	KJ190080	KJ190115	KJ190029
**Gonostomatidae**	*Sigmops bathyphilum*	MCZ 158787/KUIT 3636	Unavailable	KJ190119	KJ190033
**Gonostomatidae**	*Sigmops longipinnis*	SIO 02-47	Unavailable	KJ190118	KJ190032
**Stomiidae**	*Astronesthes formosana*	FMNH 120796	Unavailable	KJ190127	KJ190042
**Stomiidae**	*Borostomias antarcticus*	MCZ 158728/KUIT 3612	Unavailable	KJ190129	KJ190044
**Stomiidae**	*Echiostoma barbatum*	MCZ 161590/KUIT 5281	GQ860330	KJ190136	KJ190051
**Stomiidae**	*Flagellostomias boureei*	MCZ 164815/KUIT 7489	GQ860335	KJ190135	KJ190050
**Stomiidae**	*Idiacanthus antrostomus*	FMNH Uncat Field RGS6	KJ190086	KJ190128	KJ190043
**Stomiidae**	*Malacosteus niger*	MCZ 158917/KUIT 3693	Unavailable	KJ190126	KJ190041
**Stomiidae**	*Melanostomias bartonbeani*	MCZ 164153/KUIT 6514	Unavailable	KJ190125	KJ190040
**Stomiidae**	*Pachystomias microdon*	MCZ 167907/KUIT 8454	GQ860341	KJ190134	KJ190049
**Stomiidae**	*Photonectes braueri*	MCZ 164742/KUIT 7471	Unavailable	KJ190130	KJ190045
**Stomiidae**	*Photostomias guernei*	KUI 28507/KUIT 3810	GQ860353	KJ190132	KJ190047
**Stomiidae**	*Rhadinesthes decimus*	MCZ 164113/KUIT 6506	GQ860323	KJ190131	KJ190046
**Stomiidae**	*Stomias atriventer*		Unavailable	KF768166	KF768177
**Stomiidae**	*Tactostoma macropus*	KU 28258/KUIT 3238	GQ860344	KJ190133	KJ190048
**Galaxiidae**	*Aplochiton taeniatus*		JN232639	Unavailable	HQ540330
**Galaxiidae**	*Galaxias maculatus*		N232643	JX191286	AP004104
**Galaxiidae**	*Galaxiella nigrostriata*		JN232647	JX191287	AP006853
**Ateleopodidae**	*Ateleopus japonicas*		JX190838	JX191297	AP002916
**Ateleopodidae**	*Guentherus altivelis*	USNM Uncat Field CCB98-50	Unavailable	KJ190137	KJ190052
**Ateleopodidae**	*Ijimaia antillarum*		EU366725	EU366769	EU366585
**Synodontidae**	*Saurida tumbil*		KF768158	KF768164	KF768174
**Synodontidae**	*Synodus kaianus*		EU366719	EU366761	EU366578
**Aulopidae**	*Hime japonicus*		EU366687	EU366732	AB047821
**Pseudotrichonotidae**	*Pseudotrichonotus altivelis*		EU366711	EU366754	EU366570
**Paraulopidae**	*Paraulopus oblongus*		EU366709	EU366752	EU366568
**Giganturidae**	*Gigantura indica*		EU366699	EU366744	EU366557
**Bathysauridae**	*Bathysaurus ferox*		EU366689	EU366734	EU366547
**Ipnopidae**	*Bathypterois grallator*		EU366690	EU366735	EU366548
**Ipnopidae**	*Ipnops* sp.		EU366702	EU366747	EU366560
**Chlorophthalmidae**	*Chlorophthalmus nigromarginatus*	FMNH 120838	KJ190087	KJ190138	KJ190053
**Notosudidae**	*Scopelosaurus harryi*		EU366713	EU366756	EU366572
**Scopelarchidae**	*Scopelarchoides danae*	CBM-ZF T99-142	KJ190088	Unavailable	KJ190054
**Evermannellidae**	*Odontostomops normalops*		EU366706	EU366749	EU366565
**Sudidae**	*Sudis atrox*		EU366717	EU366759	EU366576
**Alepisauridae**	*Alepisaurus ferox*		EU366683	EU366729	EU366542
**Alepisauridae**	*Anotopterus pharao*		EU366686	Unavailable	GU440224
**Alepisauridae**	*Omosudis lowei*		EU366707	EU366750	EU366566
**Paralepididae**	*Lestrolepis japonica*	FMNH 120671	KJ190089	Unavailable	KJ190055
**Neoscopelidae**	*Neoscopelus macrolepidotus*		EU366727	EU366771	EU366587
**Neoscopelidae**	*Neoscopelus microchir*	FMNH 119741	KJ190098	JX191303	KF768172
**Neoscopelidae**	*Scopelegenys trista*		Unavailable	JX191304	KF768175
**Neoscopelidae**	*Solivomer arenidens*		Unavailable	Unavailable	AP012249
**Myctophidae**	*Benthosema glaciale*		KF768155	KF768160	KF768167
**Myctophidae**	*Bolinichthys longipes*	SIO 10-164	Unavailable	KJ190139	GU581197
**Myctophidae**	*Ceratoscopelus maderensis*	MCZ 159030/KUIT 3698	KJ190090	KJ190140	KJ190056
**Myctophidae**	*Diaphus dumerilii*		KF768156	KF768161	KF768169
**Myctophidae**	*Diaphus effulgens*	MCZ 159080/KUIT 3609	KJ190092	KJ190142	KJ190058
**Myctophidae**	*Diaphus theta*	KUI 27971/KUIT 2135	KJ190091	KJ190141	KJ190057
**Myctophidae**	*Diogenichthys atlanticus*	SIO 09-99	Unavailable	KJ190143	KJ190059
**Myctophidae**	*Electrona risso*	SIO 10-173	Unavailable	KJ190144	Unavailable
**Myctophidae**	*Gymnoscopelus nicholsi*		JX190845	JX191305	Unavailable
**Myctophidae**	*Hygophum reinhardtii*	SIO 09-320	Unavailable	KJ190145	Unavailable
**Myctophidae**	*Krefftichthys andersoni*		JX190846	JX191306	Unavailable
**Myctophidae**	*Lampadena speculigera*	MCZ 163213/KUIT 5916	KJ190093	KJ190146	KJ190060
**Myctophidae**	*Lampanyctus pusillus*	KUI 26890/KUIT 267	Unavailable	Unavailable	KJ190061
**Myctophidae**	*Lepidophanes guentheri*	KUI 28493/KUIT 3796	KJ190094	KJ190147	KJ190062
**Myctophidae**	*Lobianchia gemellarii*	SIO 10-171	KJ190095	KJ190148	KJ190063
**Myctophidae**	*Loweina interrupta*	CSIRO GT 6733	KJ190096	Unavailable	Unavailable
**Myctophidae**	*Myctophum obtusirostre*	FMNH 120853	KJ190097	KJ190149	KJ190064
**Myctophidae**	*Myctophum punctatum*	KUI 28499/KUIT 3802	Unavailable	KJ190150	KJ190065
**Myctophidae**	*Nannobrachium lineatum*		EU366726	EU366770	EU366586
**Myctophidae**	*Notolynchus valdiviae*	SIO 10-166	KJ190099	KJ190151	KJ190066
**Myctophidae**	*Notoscopelus caudispinosus*	MCZ 161883/KUIT 5301	KJ190100	Unavailable	KJ190067
**Myctophidae**	*Notoscopelus kroyeri*		AY430221	Unavailable	EU148278
**Myctophidae**	*Parvilux ingens*	SIO 06-90	Unavailable	Unavailable	KJ190068
**Myctophidae**	*Protomyctophum crockeri*	FMNH Uncat Field RGS6	KJ190101	KJ190152	KJ190069
**Myctophidae**	*Protomyctophum thompsanii*	KUI 27969/KUIT 2133	KJ190102	Unavailable	KJ190070
**Myctophidae**	*Scopelopsis multipunctatus*		Unavailable	Unavailable	GU805037
**Myctophidae**	*Stenobrachius leucopsaurus*	FMNH Uncat Field RGS6 T9	KJ190103	KJ190153	KJ190071
**Myctophidae**	*Symbolophorus californiensis*	FMNH Uncat Field RGS12	KJ190104	KJ190154	KJ190072
**Myctophidae**	*Taaningichthys bathyphilus*	SIO 10-174	KJ190105	KJ190155	EU148347
**Myctophidae**	*Tarletonbeania crenularis*	FMNH 122284	KJ190106	Unavailable	KJ190073
**Myctophidae**	*Triphoturus mexicanus*	FMNH 122285	KJ190107	KJ190156	KJ190074
**Polymixiidae**	*Polymixa japonica*		AY308765	EU366776	AB034826
**Zeidae**	*Zeus faber*		JX190854	Unavailable	HQ945864
**Veliferidae**	*Metavelifer multiradiatus*		EF094949	EU366772	EU366588
**Berycidae**	*Beryx decadactylus*		JX189798	Unavailable	JF492956
**Moronidae**	*Morone chrysops*		AY308767	EF032917	EU524141

Supplementary Table 2: Lanternfish specimens examined for photophore position and placement.

Genus	Species	Institution	Catalog Number
Diaphus	*brachycephalus*	MCZ	121432
Diaphus	*mollis*	MCZ	90306
Diaphus	*holti*	MCZ	12062
Diaphus	*parri*	MCZ	151451
Diaphus	*holti*	MCZ	120623
Diaphus	*brachycephalus*	MCZ	121662
Diaphus	*dumerilii*	MCZ	120885
Diaphus	*problematicus*	MCZ	128058
Diaphus	*leutkeni*	MCZ	120148
Diaphus	*termophilus*	MCZ	118161
Diaphus	*termophilus*	MCZ	118159
Diaphus	*perspicillatus*	MCZ	126693
Diaphus	*garmani*	MCZ	151630
Diaphus	*garmani*	MCZ	90863
Gonichthys	*barnesi*	MCZ	103190
Gonichthys	*cocco*	MCZ	116669
Gonichthys	*tenuiculus*	MCZ	103199
Myctophum	*spinosus*	MCZ	151450
Myctophum	*asperum*	MCZ	106460
Myctophum	*fissunovi*	MCZ	81734
Myctophum	*obtusirostre*	MCZ	51389
Myctophum	*obtusirostre*	MCZ	105868
Myctophum	*nitidulus*	MCZ	157588
Centrobranchus	*nigroocellatus*	MCZ	98844
Protomyctophum	*subparallelum*	MCZ	102557
Symbolophorus	*rufinus*	MCZ	103536
Symbolophorus	*rufinus*	MCZ	148934
Symbolophorus	*boops*	MCZ	103574
Symbolophorus	*boops*	MCZ	103573
Symbolophorus	*evermanni*	MCZ	148720
Symbolophorus	*evermanni*	MCZ	148717
Symbolophorus	*veranyi*	MCZ	45333
Symbolophorus	*veranyi*	MCZ	111606
Protomyctophum	*arcticum*	MCZ	102601
Hygophum	*macrochir*	MCZ	115225
Hygophum	*macrochir*	MCZ	115290
Hygophum	*taaningi*	MCZ	11451
Hygophum	*benoiti*	MCZ	116153
Hygophum	*reinhardtii*	MCZ	114759
Hygophum	*hygomii*	MCZ	115383
Hygophum	*hygomii*	MCZ	115725
Stenobrachius	*leucopsarus*	MCZ	88957
Ceratoscopelus	*maderensis*	MCZ	166121
Ceratoscopelus	*maderensis*	MCZ	100705
Ceratoscopelus	*warmingi*	MCZ	92411
Gymnoscopelus	*braueri*	MCZ	148792
Gymnoscopelus	*braueri*	MCZ	148797
Notoscopelus	*caudispinous*	MCZ	157882
Notoscopelus	*elongatus kroyeri*	MCZ	104150
Notoscopelus	*elongatus kroyeri*	MCZ	104147
Notoscopelus	*bolini*	MCZ	103988
Lepidophanes	*guentheri*	MCZ	108541
Lampanyctus	*crocodilus*	MCZ	55470
Lampanyctus	*iselinoides*	MCZ	102845
Lampanyctus	*australis*	MCZ	55034
Lampanyctus	*mexicanus*	MCZ	45398
